# Research on collaborative innovation optimization strategies for digitally enabled higher education ecosystems

**DOI:** 10.1371/journal.pone.0302285

**Published:** 2024-04-18

**Authors:** Yan Zhao, Zheng Yang

**Affiliations:** 1 Personnel Department, Shenyang Institute of Engineering, Shenyang, China; 2 Faculty Affairs Office of the Shenyang Institute of Engineering Committee, Shenyang Institute of Engineering, Shenyang, China; Zhejiang University of Technology, CHINA

## Abstract

Digitally enabled higher education involves the in-depth use of new-generation digital technology, which has subverted and innovated the traditional teaching mode, driven the development of high-quality teaching and learning, and improved teachers’ teaching experience, and increased efficiency. Based on ecosystem theory, this paper constructs a higher education ecosystem with the government, enterprises, and universities as the core participating subjects. It considers the participating subjects’ effort level and the ecosystem’s overall benefits under the three scenarios of noncooperative research and development (R&D), cost sharing, and cooperative R&D. The results show that (1) the service innovation effort level of the three parties increases with increasing human resource level and technology maturity, and the government’s benefit decreases with increasing cost of fulfilling social responsibility. (2) The government’s cost subsidies to universities and enterprises can enhance the service innovation level of both parties and increase the optimal returns of the three parties and the ecosystem as a whole. (3) In the cooperative R&D game scenario, the effort level of the three parties and the total ecosystem returns are greater than those in the noncollaborative R&D scenario, and after determining the subsidy coefficients of the government, Pareto optimality of the three parties and the ecosystem as a whole can be achieved. The conclusions of this study can aid in understanding the dynamic evolution mechanism of digitally enabled higher education and provide a realistic decision-making reference for higher education ecosystem managers.

## Introduction

Digitally enabled higher education is a contemporary response to the development of the digital era, an inevitable choice to promote the modernization of education, and a sure way to achieve fair and quality education. Accompanied by the acceleration of the new round of scientific and technological revolution and industrial change, the new generation of digital technologies represented by artificial intelligence and big data drove the generation and development of digital transformation in higher education. From the perspective of economics, higher education, as an activity with public attributes [1], is a social activity that is used to cultivate high-quality talent and serve economic and social development in the digital era. Moreover, digitalization is the key to the high-quality development of higher education, effectively integrating the participating elements in the development process and decentralizing the formation of a higher education innovation ecosystem by breaking time and space constraints [2]. The core concept of the innovation ecosystem was first introduced by the US President’s Council of Advisors on Science and Technology in early 2004, emphasizing that the innovation ecosystem is a powerful tool that is needed for the US economy to prosper and maintain its leadership position in the global economy, with the strength of the nation’s skills in the fields of science, technology, engineering, and math as a central driver [3]. A higher education innovation ecosystem is the expansion of the innovation ecosystem in the field of higher education, reflecting the two essential features of higher education element intervention and the ecological system, and its connotation is the gradual penetration of higher education in the innovation ecosystem and the complex adaptive system constituted by mutual promotion and in-depth integration of the two. The digitally enabled higher education innovation ecosystem emphasizes the synergistic and symbiotic relationship between higher education subjects due to the reorganization of elements and the logical change of systematic behaviors due to the introduction of digital elements. Moreover, digitally enabled higher education innovation ecosystems reflect the logical and deterministic relationships among innovation subjects, technology, and social environments, in which highly efficient teachers and students take the initiative to adapt to the social environment and make full use of digital technology; the development of digital technology reacts to the social environment and helps to improve the quality of higher education; and the social environment plays a role in digital technology and creates a favorable learning atmosphere for teachers and students [4].

At present, multibody collaborative innovation in innovation ecosystems is the new paradigm of today’s international technological innovation. The flow of innovative resources catalyzes the establishment of synergistic relationships, the flow of creative resources in the process of new technology, and new knowledge of multiple iterations to achieve technological innovation; the process of government macrocontrol can promote the transformation of knowledge [5]. In recent years, many scholars have confirmed through research that multibody collaborative cooperation is conducive to the research development and transformation of scientific and technological achievements and subsequent technological innovation. For example, Chen et al. constructed a game model to explore the strategic evolutionary path in the process of systematic innovation in local engineering colleges and universities and proposed that collaborative innovation is a long-term evolutionary process [6]. In addition, Mauri et al. found that in the higher education system, task complexity, the subject’s technological maturity, and effective feedback are important influencing factors in promoting system innovation [7]. Therefore, dissecting the optimization strategies of multiparty participation in collaborative innovation in higher education innovation ecosystems and exploring the drivers of specific influences are crucial to achieving stable development of higher education innovation ecosystems.

The literature has described specific processes combining and pioneering theoretical research on constructing and generating innovation ecosystems and digitally enabled higher education. Nevertheless, more systematic and targeted research is needed on collaborative multibody innovation’s evolutionary mechanisms and optimization strategies. First, the existing research describes the construction process of a higher innovation ecosystem system and reveals the motivation of the relevant subjects to construct and participate in the ecosystem but lacks a systematic analysis of the specific behaviors of the members therein, such as the technological maturity of the innovation subjects and the level of their efforts in fulfilling their social responsibilities. Second, studies from the complexity science perspective have qualitatively analyzed the conditions and mechanisms of forming the collaborative innovation process but have yet to reveal the nonlinear interaction mechanisms of the participating subjects in the system. Here, existing studies are mainly based on case studies and theoretical analyses, and a more robust method is needed to explore the interaction mechanism of different collaborative innovation scenarios in depth. For this reason, based on existing research, this paper provides a theoretical analysis of the collaborative innovation of members in higher education innovation ecosystems, constructs decision-making models of three scenarios using differential game methods, and simulates and analyses them through numerical simulation to derive the specific impact effects of different influencing factors.

The main innovations of this paper are as follows: (1) based on innovation ecosystem theory, we constructed a higher education ecosystem, innovatively bypassed the linear innovation paradigm to the nonlinear innovation paradigm in higher education, and analyzed the benefits of the higher education ecosystem in different scenarios from the perspective of dynamic decision-making; (2) combined with the reality of the development of higher education, we categorized collaborative innovation in the higher education ecosystem into three innovation scenarios, namely, noncollaborative R&D, cost sharing, and collaborative cooperation, and constructed game models for comparative analysis; and (3) introduced critical variables such as the effort cost coefficient and technology maturity coefficient in the decision-making system and precisely analyzed the influence of critical variables through numerical simulation and verified the effectiveness of the model.

## Theoretical basis and literature review

### Digitally enabled

Digitally enabled refers to empowering specific groups of people through digital tools such as big data, the internet, and artificial intelligence to obtain the corresponding life skills and survival ability [8]. In the era of the rapid development of science and technology, digital enabling, as a prominent driving force of science and technology, has led to transformation and innovation in various fields. In higher education, digital access has demonstrated great potential. With the development of modern information technology, traditional education methods are also changing, and the application of technology has become an indicator of the development and innovation of teaching methods [9]. Current scholars focus on how digital technology and education can be better combined. The practical application of digital technology in the classroom is the main issue; recent research has focused on using digital devices and intelligent technology in schools rather than relying on digital technology to flexibly change teaching methods [10]. Nikou established a conceptual model to test the effect of digital technology on the literacy ability of college teachers and students [11], and Sholikah empirically examined the impact of the use of digital technology in education on student satisfaction and how this impact occurs after using a population sampling method on university students in Indonesia [12].

Habibi empirically examined the impact of digital technology on the effectiveness of teaching and learning when making educational changes by using students in Indonesia, where students’ access to digital technology determines the efficacy of educational changes [13]. Teachers, as the main educator of students, significantly affect the application of digital technology. Maksimovic analyzed physical education teachers in digital technology to support the need to improve educational competence and studied how to apply digital technology to classroom teaching. Their findings showed that the digital competence of teachers is essential [14]. When the digital technology reform of the education system was fully implemented in Sweden, Karlsson embarked on a study of teachers’ evaluation of the use of digital technology. Among the many influencing factors, curriculum development plays an important role, but the role of factors such as digital equipment, areas of knowledge, and students’ needs should not be ignored [15]. Oliveira examined the use of digital technology in Brazil during the neo-Kwanzaa outbreak in primary education, and by analyzing, among other things, policy documents, he argued that students’ use of digital technology depended on family income. Therefore, regional and geographic inequities in education emerged, exacerbated by the lack of government policies for providing digital resources [16]. Rafalow similarly examined the issue of inequality in education due to the use of digital technology, with schools as the providers of educational resources determining the kind of help provided to students, and the use of digital technologies in teaching and learning has allowed this categorization function of schools to exacerbate inequalities inside and outside the classroom [17].

### Higher education ecosystem

The concept of an "ecosystem" was initially proposed by Tansler, a British scholar, and is based on three basic assumptions: first, the aggregation of the components of the system is based on shared values; second, the elements play different roles and perform heterogeneous functions in the system; and third, the components are interdependent and coevolve to reach a balanced system state [18]. The higher education ecosystem is a subsystem of society that structurally includes the main body, decomposers, the environment, etc., of which producers mainly refer to various higher education institutions [19]. Current research in academia primarily focuses on innovation and entrepreneurship in higher education ecosystems, and innovation and entrepreneurship systems should be constructed around universities, linking multiple production factors to cultivate innovative talent [20]. The entrepreneurship education ecosystem should be based on integrating entrepreneurship and education, highlighting key features such as interconnection in the construction of the system and relying on the system to realize the production of knowledge and the creation of value [21]. Sun analyzed the innovation ecosystem of higher education from the culture of innovation and proposed a strategy for constructing the innovation education system from the culture perspective [22].

The higher education ecosystem is crossing from partial digitalization to high digitalization, the linkage between the elements in the ecosystem is changing, the learning of the subject of the education ecosystem is moving toward personalization, the cooperation of the type of education ecosystem is becoming more diverse, the development of the education ecosystem region is moving toward balancing, and the crossing of the level of the education ecosystem is becoming more precise [23]. Relying on digital technology to make changes to the educational ecosystem has recently become a concern of scholars. Abney analyzed the impact of integrating social media into teaching, arguing that social media provides a new learning environment for the educational ecosystem, and the results of its empirical analysis proved the effectiveness of this method [24]. The integration of e-learning in the teaching-learning process has led to a change in the educational approach, and Rodrigues approached this from the perspective of the scholarly ecosystem and used methods such as interviews to identify the structures that influence the adoption of e-learning in the higher education ecosystem in Brazil [25].

### Differential game theory

The earliest study of differential game theory can be traced back to the mid-20th century. Scholars combined differential equations and game theory in this period to study the impact of dynamic changes in participants’ strategies on the evolution of the system. With the development of computer technology, scholars used numerical methods and simulation techniques to conduct more in-depth research on the complex behaviors of differential games, and the theory of differential games has been gradually applied to many fields, such as economics, management and engineering, management and engineering. It has been used to study market competition, resource allocation, simulation evolution, etc. I developed a differential game controller in the analysis of human-robot interaction, which is used to help the robot immediately recognize the user’s control strategy to acknowledge the motion state better and respond to it [26]. Franceschi constructed a role arbitration framework for the human-robot role arbitration problem based on differential game theory by referring to cooperative game theory. The arbitration problem refers to the modeling of unified game theory to simulate and analyze the problem [27]. Yang explored the optimal strategies of the government and enterprises under market-driven and government-driven modes based on differential game theory and constructed a courier packaging waste recycling system with the joint participation of the government, individuals, and enterprises [28].

### Gaps and insights in the existing literature

The application of digital technology in higher education provides unprecedented opportunities for building more flexible and efficient educational ecosystems. However, in the in-depth study of digitally enabled higher education ecosystems, we find that there are some significant shortcomings in the literature: (1) Current research on digitally enabled higher education ecosystems focuses on the application of technology and empirical analysis but lacks an in-depth understanding of dynamic evolution and optimization strategies, and many studies have focused on the specific tools and platforms of digital technologies while ignoring the long-term impacts of these technologies on ecosystems in different periods and contexts. Many studies focus on particular tools and platforms of digital technologies but neglect the long-term impacts of these technologies on ecosystems in different periods and contexts. The lack of systematic theoretical research on the dynamic evolution mechanism of higher education ecosystems also leads to deficiencies in developing optimization strategies. (2) Most of the literature does not consider the dynamic and continuous characteristics of the innovation and decision-making process of higher education services, nor does it accurately portray the dynamic game that continues to be played over a continuous period, which also omits the critical factors in the dynamic evolution and decision-making process of higher education. (3) At present, game theory-based pedagogical research focuses on the impact of different strategic choices between subjects on the process of educational evolution, few scholars pay attention to the differentiated impact of critical factors on the same strategic choices, and there is a lack of validation of the effectiveness of the model.

Based on this, this paper constructs a dynamic evolution and decision-making model for the collaborative development of higher education ecosystems in the context of digitization with the help of differential game theory, compares the equilibrium structure and decision-making effect under different scenarios, identifies the guiding direction and intensity of higher education ecosystem service innovation, and finally verifies the effectiveness of the model through numerical simulation to provide a theoretical basis for effectively guiding the high-quality development of higher education.

## Problem description and modeling assumptions

### Problem description

**Fig 1:**
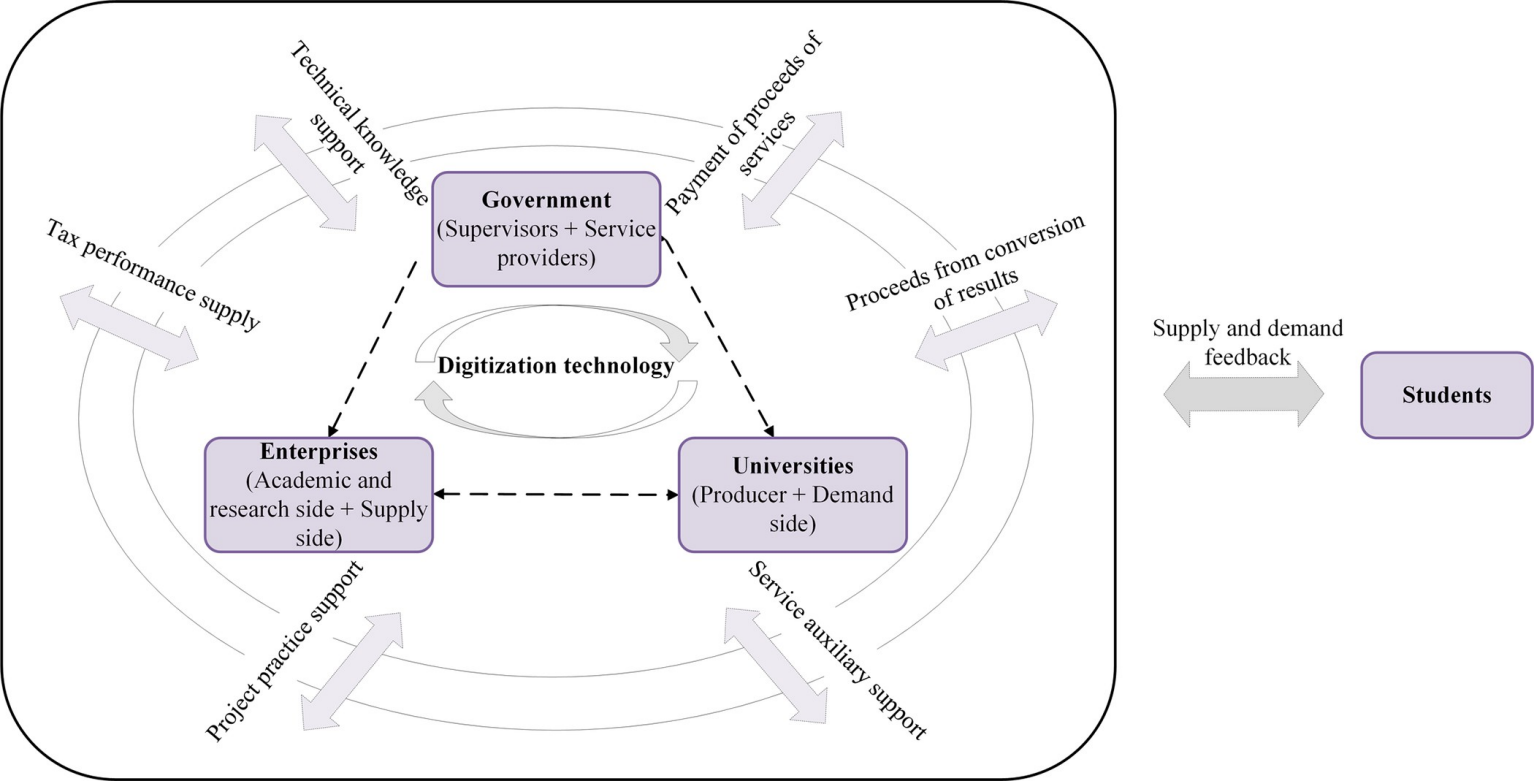
Mechanism map of the functioning of the higher education ecosystem.

The higher education innovation ecosystem is the expansion of the innovation ecosystem in the context of higher education, and it is a complex dynamic system composed of industrial subjects related to higher education innovation based on synergy and competition. Accompanied by the rise and application of several new digital technologies, such as artificial intelligence, big data, blockchain, and cloud computing, the collaborative innovation relationship in the higher education innovation ecosystem has been gradually reconstructed, which in turn continuously generates a sharing mechanism and realizes the value added to higher education. Then, this paper investigates the issue of collaborative R&D among different actors within the higher education ecosystem to improve product and service innovation in the context of digitization, focusing on the impact of different collaboration models and incentive strategies on the participating actors and the ecosystem. The conceptual model constructed in this paper is shown in Fig 1. In a higher education ecosystem composed of enterprises (*E*), universities (*U*) and governments (*G*), enterprises, as the research and supply side, carry out basic research on education technology; universities, as the production and demand side, carry out the development of education technology; and the government, as the supervisor and service provider, is committed to promoting the collaborative innovation research and development process of the higher education ecosystem in the context of digitization. The three main parties are independent of each other, and there is cooperation; that is, enterprises independently carry out independent research and development. On the other hand, through university cooperation in research and development, universities, in addition to complementary resource docking with core enterprises, also upgrade, to a certain extent, their own core technology research and development. To encourage enterprises to carry out research and develop core technologies and to promote the development of the economy and the market, the government, on the one hand, provides subsidies to enterprises and colleges and universities that carry out technological research and development and, on the other hand, rewards those enterprises and colleges and universities that take the initiative to fulfill their social responsibilities.

### Modeling assumptions

**Hypothesis 1:** The innovation costs of enterprises (*E*), universities (*U*) and the government (*G*) are convex with their respective innovation inputs; as the innovation inputs of each subject increase, the costs to be paid will increase, and thus, the innovation costs of each subject at moment *t* can be expressed as follows:

$$C_{E}(t)=(\frac{\mu_{E}}{2}+\frac{1}{k_{E}})E_{E}^{2}(t);$$

$$C_{U}(t)=(\frac{\mu_{U}}{2}+\frac{1}{k_{U}})E_{U}^{2}(t);$$

$$C_{G}(t)=\frac{1}{2}(\mu_{G}+\nu_{G})E_{G}^{2}(t)$$ (1)

In Eq (1), *μ*_*i*_(*i* = *E*,*U*,*G*) represents the coefficient of the cost of effort of the participant to carry out innovative research and development, *k*_*i*_(*i* = *E*,*U*) represents the coefficient of technological familiarity of the participant, and *v*_*G*_ represents the coefficient of the cost of effort of the government in fulfilling its social responsibility. *E*_*E*_(*t*), *E*_*U*_(*t*), and *E*_*G*_(*t*) represent the innovation endeavors of enterprises, universities and governments, respectively, at moment *t*.

**Hypothesis 2:** The technological level of the higher education ecosystem at moment *t* is jointly determined by the innovation inputs from enterprises, universities and the government; the greater the innovation inputs from each subject are, the greater the technological level of the higher education ecosystem. In addition, the technology level will slowly decrease over time.


$$\left\{ \begin{array}{l} W^{'}(t)=\frac{dW(t)}{dt}=\eta_{E}E_{E}(t)+\eta_{U}E_{U}(t)+\eta_{G}E_{G}(t)-\delta W(t)\\ W(0)=W_{0}\geq 0 \end{array}\right.$$ (2)

**Hypothesis 3:** Innovation inputs from enterprises, universities and the government at the moment *t* directly affect the total returns of the higher education ecosystem. At the same time, improving technology levels will also indirectly affect the total return of the higher education ecosystem by enhancing its overall competitiveness. Assume that the total return of technological innovation in the higher education innovation ecosystem at moment *t* is:

$$\pi (t)=\lambda_{E}E_{E}(t)+\lambda_{U}E_{U}(t)+\lambda_{G}E_{G}(t)+\theta W(t)$$ (3)

**Hypothesis 4:** All actors in a higher education ecosystem make rational decisions based on complete information. they all aim to maximize their returns and have the same discount rate *ρ*. The total benefits are distributed among enterprises, universities and the government, assuming that enterprises receive *ω*_*E*_, universities receive *ω*_*U*_, and the government receives 1‒*ω*_*E*_ ‒*ω*_*U*_ (0<*ω*_*E*_,*ω*_*U*_,1‒*ω*_*E*_‒*ω*_*U*_<1), and the distribution ratio is determined by prior negotiation based on the contributions of each subject. At the same time, to promote the development of technological coinnovation in the higher education ecosystem in the context of digitization, the government subsidizes the cost of innovation for enterprises and universities, assuming that the subsidy rates are *α* and *β* (0≤*α*, *β*≤1), respectively. The objective functions of enterprises, universities and the government are as follows:

$$\underset{E_{E}(t)}{\max}J_{E}=\int_{0}^{\infty }e^{-\rho t}[\omega_{E}\pi (t)+(\alpha (t)-1)(\frac{\mu_{E}}{2}+\frac{1}{k_{E}})E_{E}^{2}(t)]dt$$ (4)

$$\underset{E_{U}(t)}{\max}J_{U}=\int_{0}^{\infty }e^{-\rho t}[\omega_{U}\pi (t)+(\beta (t)-1)(\frac{\mu_{U}}{2}+\frac{1}{k_{U}})E_{U}^{2}(t)]dt$$ (5)

$$\begin{array}{l} \underset{E_{G}(t)}{\max}J_{G}=\int_{0}^{\infty }e^{-\rho t}[(1-\omega_{E}-\omega_{U})\pi (t)-\frac{1}{2}(\mu_{G}+\nu_{G})E_{G}^{2}(t)-\alpha (t)(\frac{\mu_{E}}{2}+\frac{1}{k_{E}})E_{E}^{2}(t)-\\ \beta (t)(\frac{\mu_{U}}{2}+\frac{1}{k_{U}})E_{U}^{2}(t)]dt \end{array}$$ (6)

The model contains control variables *E*_*E*_(*t*), *E*_*U*_(*t*), *E*_*G*_(*t*), *α* and *β* and state variables *W*(*t*) Since it is impossible to solve the model under dynamic parameter conditions, it is assumed that all parameters in this paper are time-independent positive constants, and the time variable *t* will be omitted later for computational convenience.

In summary, the symbols and meanings of the parameters involved in the model are shown in Table 1.

**Table 1:** Parameter symbols and descriptions.

Symbol	Description
*μ*_*i*_	Coefficient of cost of efforts of participating agents to carry out innovative R&D, *i* = *E*,*U*,*G*.
*k*_*i*_	Coefficient of technical familiarity of participating subjects, *i* = *E*,*U*.
*v*_*G*_	Coefficient of cost of efforts made by the Government to fulfill its social responsibilities.
*E*_*E*_(*t*)	The level of corporate innovation efforts at moment t.
*E*_*U*_(*t*)	The level of innovation endeavor in universities at moment t.
*E*_*G*_(*t*)	The level of government innovation efforts at moment t.
*W*(*t*)	Technology level of higher education ecosystems at moment t.
*W*(0)	Initial level of technological innovation.
*η*_*i*_	Coefficient of influence of the level of innovation efforts of participating subjects on the technological level of the higher education ecosystem, *i* = *E*,*U*,*G*.
*δ*	Decay rate of technology levels in higher education ecosystems over time.
*π*(*t*)	Total ecosystem benefits from higher education at moment t.
*λ*_*i*_	The extent to which the level of effort affects the total ecosystem benefits of higher education, *i* = *E*,*U*,*G*.
*θ*	Extent of impact of innovation on total returns.
*ω*_*E*_	Earnings distribution coefficients for enterprises.
*ω*_*U*_	Benefit-sharing coefficients for higher education institutions.
1‒*ω*_*E*_‒*ω*_*U*_	Government revenue-sharing coefficients.
*α*	Rate of government subsidies to firms for innovation costs.
*β*	Ratio of government subsidies to universities for innovation costs.

## Model construction

### Noncooperative research and development model

In the noncooperative R&D model, cooperation among the parties involved in the higher education ecosystem is loose and only formal. Enterprises, universities and the government all independently choose their own research and development efforts based on equal status to maximize their benefits, and the optimal strategy of the three parties at this time is the Nash equilibrium strategy with static feedback. At the same time, the government will not share the R&D costs of enterprises and universities under rational conditions *α* = *β* = 0.

To obtain the equilibrium strategy of the problem, the Hamilton–Jacobi–Bellman equation (hereafter referred to as the HJB equation) is used to solve the problem according to the sufficient conditions of Nash equilibrium with static feedback. The innovation return functions of enterprises, universities and the government are *V*_*E*_(*W*), *V*_*U*_(*W*), and *V*_*G*_(*W*), respectively, which satisfy the following HJB equation:

$$\rho V_{E}(W)=\underset{E_{E}\geq 0}{\max}\{\omega_{E}\pi (t)-(\frac{\mu_{E}}{2}+\frac{1}{k_{E}})E_{E}^{2}+V_{E}^{'}(W)(\eta_{E}E_{E}+\eta_{U}E_{U}+\eta_{G}E_{G}-\delta W)\}$$ (7)

$$\rho V_{U}(W)=\underset{E_{U}\geq 0}{\max}\{\omega_{U}\pi (t)-(\frac{\mu_{U}}{2}+\frac{1}{k_{U}})E_{U}^{2}+V_{U}^{'}(W)(\eta_{E}E_{E}+\eta_{U}E_{U}+\eta_{G}E_{G}-\delta W)\}$$ (8)

$$\rho V_{G}(W)=\underset{E_{G}\geq 0}{\max}\{(1-\omega_{E}-\omega_{U})\pi (t)-\frac{1}{2}(\mu_{G}+\nu_{G})E_{G}^{2}+V_{G}^{'}(W)(\eta_{E}E_{E}+\eta_{U}E_{U}+\eta_{G}E_{G}-\delta W)\}$$ (9)

The right sides of Eq (7)–Eq (9) are obtained by taking the first-order partial derivatives of Eq (7)–Eq (9) for *E*_*E*_, *E*_*U*_, and *E*_*G*_, respectively, and setting them equal to zero:

$$E_{E}=\frac{k_{E}[\omega_{E}\lambda_{E}+\eta_{E}V_{G}^{'}(W)]}{\mu_{E}k_{E}+2},$$

$$E_{U}=\frac{k_{U}[\omega_{U}\lambda_{U}+\eta_{U}V_{U}^{'}(W)]}{\mu_{U}k_{U}+2},$$

$$E_{G}=\frac{k_{G}[(1-\omega_{E}-\omega_{U})\lambda_{G}+\eta_{G}V_{G}^{'}(W)]}{\mu_{G}+\nu_{G}}$$ (10)

Substituting Eq (10) into the HJB equation and collapsing gives:

$$\begin{array}{l} \rho V_{E}(W)=[\omega_{E}\theta -\delta V_{E}^{'}(W)]W+\frac{k_{E}{\left[\omega_{E}\lambda_{E}+\eta_{E}V_{E}^{'}(W)\right]}^{2}}{2(\mu_{E}k_{E}+2)}\\ +\frac{\left[\omega_{E}\lambda_{U}+\eta_{U}V_{E}^{'}(W)]k_{U}[\omega_{U}\lambda_{U}+\eta_{U}V_{U}^{'}(W)\right]}{\mu_{U}k_{U}+2}+\frac{\left[\omega_{E}\lambda_{G}+\eta_{G}V_{E}^{'}(W)][(1-\omega_{E}-\omega_{U})\lambda_{G}+\eta_{G}V_{G}^{'}(W)\right]}{\mu_{G}+\nu_{G}} \end{array}$$ (11)

$$\begin{array}{l} \rho V_{U}(W)=[\omega_{U}\theta -\delta V_{U}^{'}(W)]W+\frac{\left[\omega_{U}\lambda_{E}+\eta_{E}V_{U}^{'}(W)]k_{E}[\omega_{E}\lambda_{E}+\eta_{E}V_{E}^{'}(W)\right]}{\mu_{E}k_{E}+2}\\ +\frac{k_{U}{\left[\omega_{U}\lambda_{U}+\eta_{U}V_{U}^{'}(W)\right]}^{2}}{2(\mu_{U}k_{U}+2)}+\frac{\left[\omega_{U}\lambda_{G}+\eta_{G}V_{U}^{'}(W)][(1-\omega_{E}-\omega_{U})\lambda_{G}+\eta_{G}V_{G}^{'}(W)\right]}{\mu_{G}+\nu_{G}} \end{array}$$ (12)

$$\begin{array}{l} \rho V_{G}(W)=[(1-\omega_{E}-\omega_{U})\theta -\delta V_{G}^{'}(W)]W+\frac{\left[(1-\omega_{E}-\omega_{U})\lambda_{E}+\eta_{E}V_{G}^{'}(W)]k_{E}[\omega_{E}\lambda_{E}+\eta_{E}V_{E}^{'}(W)\right]}{\mu_{E}k_{E}+2}\\ +\frac{\left[(1-\omega_{E}-\omega_{U})\lambda_{U}+\eta_{U}V_{G}^{'}(W)]k_{U}[\omega_{U}\lambda_{U}+\eta_{U}V_{U}^{'}(W)\right]}{\mu_{U}k_{U}+2}+\frac{{\left[(1-\omega_{E}-\omega_{U})\lambda_{G}+\eta_{G}V_{G}^{'}(W)\right]}^{2}}{2(\mu_{G}+\nu_{G})} \end{array}$$ (13)

According to Eq (11)—Eq (13), the linear optimal functions *V*_*E*_(*W*) = *f*_1_*W*+*f*_2_, *V*_*U*_(*W*) = *h*_1_*W*+*h*_2_, and *V*_*G*_(*W*) = *k*_1_*W*+*k*_2_ satisfy the above equation, where *f*_1_*f*_2_, *h*_1_, *h*_2_, *k*_1_, *k*_2_, and are constants. Substituting *V*_*E*_(*W*), *V*_*U*_(*W*), *V*_*G*_(*W*) and their first-order derivatives with respect to *W* gives:

$$\begin{array}{l} \rho (f_{1}W+f_{2})=(\omega_{E}\theta -\delta f_{1})W+\frac{k_{E}{\left(\omega_{E}\lambda_{E}+\eta_{E}f_{1}\right)}^{2}}{2(\mu_{E}k_{E}+2)}\\ +\frac{\left(\omega_{E}\lambda_{U}+\eta_{U}f_{1})k_{U}(\omega_{U}\lambda_{U}+\eta_{U}h_{1}\right)}{\mu_{U}k_{U}+2}+\frac{\left(\omega_{E}\lambda_{G}+\eta_{G}f_{1})[(1-\omega_{E}-\omega_{U})\lambda_{G}+\eta_{G}k_{1}\right]}{\mu_{G}+\nu_{G}} \end{array}$$ (14)

$$\begin{array}{l} \rho (h_{1}W+h_{2})=(\omega_{U}\theta -\delta h_{1})W+\frac{\left(\omega_{U}\lambda_{E}+\eta_{E}h_{1})k_{E}(\omega_{E}\lambda_{E}+\eta_{E}f_{1}\right)}{\mu_{E}k_{E}+2}\\ +\frac{k_{U}{\left(\omega_{U}\lambda_{U}+\eta_{U}h_{1}\right)}^{2}}{2(\mu_{U}k_{U}+2)}+\frac{\left(\omega_{U}\lambda_{G}+\eta_{E})[(1-\omega_{E}-\omega_{U})\lambda_{G}+\eta_{G}k_{1}\right]}{\mu_{G}+\nu_{G}} \end{array}$$ (15)

$$\begin{array}{l} \rho (k_{1}W+k_{2})=[(1-\omega_{E}-\omega_{U})\theta -\delta k_{1}]W+\frac{\left[(1-\omega_{E}-\omega_{U})\lambda_{E}+\eta_{E}k_{1}]k_{E}(\omega_{E}\lambda_{E}+\eta_{E}f_{1}\right)}{\mu_{E}k_{E}+2}\\ +\frac{\left[(1-\omega_{E}-\omega_{U})\lambda_{U}+\eta_{U}k_{1}]k_{U}(\omega_{U}\lambda_{U}+\eta_{U}h_{1}\right)}{\mu_{U}k_{U}+2}+\frac{{\left[(1-\omega_{E}-\omega_{U})\lambda_{G}+\eta_{G}k_{1}\right]}^{2}}{2(\mu_{G}+\nu_{G})} \end{array}$$ (16)

The parameter values of the optimal return function are found to be:

$$f_{1}^{N}=\frac{\omega_{E}\theta }{\rho +\delta },\;h_{1}^{N}=\frac{\omega_{U}\theta }{\rho +\delta },\;h_{1}^{N}=\frac{(1-\omega_{E}-\omega_{U})\theta }{\rho +\delta }$$ (17)

$$\begin{array}{l} f_{2}^{N}=\frac{\omega_{E}^{2}k_{E}{\left[\lambda_{E}(\rho +\delta )+\theta \eta_{E}\right]}^{2}}{2(\mu_{E}k_{E}+2)\rho {\left(\rho +\delta \right)}^{2}}+\frac{\omega_{E}\omega_{U}k_{U}{\left[\lambda_{U}(\rho +\delta )+\theta \eta_{U}\right]}^{2}}{(\mu_{U}k_{U}+2)\rho {\left(\rho +\delta \right)}^{2}}+\\ \frac{\omega_{E}(1-\omega_{E}-\omega_{U}){\left[\lambda_{G}(\rho +\delta )+\theta \eta_{G}\right]}^{2}}{(\mu_{G}+\nu_{G})\rho {\left(\rho +\delta \right)}^{2}} \end{array}$$ (18)

$$\begin{array}{l} h_{2}^{N}=\frac{\omega_{E}\omega_{U}k_{E}{\left[\lambda_{E}(\rho +\delta )+\theta \eta_{E}\right]}^{2}}{(\mu_{E}k_{E}+2)\rho {\left(\rho +\delta \right)}^{2}}+\frac{\omega_{U}^{2}k_{U}{\left[\lambda_{U}(\rho +\delta )+\theta \eta_{U}\right]}^{2}}{2(\mu_{U}k_{U}+2)\rho {\left(\rho +\delta \right)}^{2}}+\\ \frac{\omega_{U}(1-\omega_{E}-\omega_{U}){\left[\lambda_{G}(\rho +\delta )+\theta \eta_{G}\right]}^{2}}{(\mu_{G}+\nu_{G})\rho {\left(\rho +\delta \right)}^{2}} \end{array}$$ (19)

$$\begin{array}{l} k_{2}^{N}=\frac{\omega_{E}(1-\omega_{E}-\omega_{U})k_{E}{\left[\lambda_{E}(\rho +\delta )+\theta \eta_{E}\right]}^{2}}{(\mu_{E}k_{E}+2)\rho {\left(\rho +\delta \right)}^{2}}+\frac{\omega_{U}(1-\omega_{E}-\omega_{U})k_{U}{\left[\lambda_{U}(\rho +\delta )+\theta \eta_{U}\right]}^{2}}{(\mu_{U}k_{U}+2)\rho {\left(\rho +\delta \right)}^{2}}+\\ \frac{{\left(1-\omega_{E}-\omega_{U}\right)}^{2}{\left[\lambda_{G}(\rho +\delta )+\theta \eta_{G}\right]}^{2}}{2(\mu_{G}+\nu_{G})\rho {\left(\rho +\delta \right)}^{2}} \end{array}$$ (20)

Substituting $V_{E}^{'}(W)=f_{1}^{N}$, $V_{U}^{'}(W)=h_{1}^{N}$, and $V_{G}^{'}(W)=k_{1}^{N}$ into the HJB equation set, the optimal level of innovation input effort for each of the static feedback Nash equilibrium strategies of enterprises, universities and the government under the noncooperative R&D model is obtained:

$$E_{E}^{N}=\frac{\omega_{E}k_{E}[\lambda_{E}(\rho +\delta )+\theta \eta_{E}]}{\left(\mu_{E}k_{E}+2)(\rho +\delta \right)},$$

$$E_{U}^{N}=\frac{\omega_{U}k_{U}[\lambda_{U}(\rho +\delta )+\theta \eta_{U}]}{\left(\mu_{U}k_{U}+2)(\rho +\delta \right)},$$

$$E_{G}^{N}=\frac{\left(1-\omega_{E}-\omega_{U})[\lambda_{G}(\rho +\delta )+\theta \eta_{G}\right]}{\left(\mu_{G}+\nu_{G})(\rho +\delta \right)}$$ (21)

By substituting the solved parameter values into the optimal benefit functions of the firm, the university and the government are collated:

$$\begin{array}{l} V_{E}^{N}(W)=\frac{\omega_{E}\theta }{\rho +\delta }W+\frac{\omega_{E}^{2}k_{E}{\left[\lambda_{E}(\rho +\delta )+\theta \eta_{E}\right]}^{2}}{2(\mu_{E}k_{E}+2)\rho {\left(\rho +\delta \right)}^{2}}+\frac{\omega_{E}\omega_{U}k_{U}{\left[\lambda_{U}(\rho +\delta )+\theta \eta_{U}\right]}^{2}}{(\mu_{U}k_{U}+2)\rho {\left(\rho +\delta \right)}^{2}}+\\ \frac{\omega_{E}(1-\omega_{E}-\omega_{U}){\left[\lambda_{G}(\rho +\delta )+\theta \eta_{G}\right]}^{2}}{(\mu_{G}+\nu_{G})\rho {\left(\rho +\delta \right)}^{2}} \end{array}$$ (22)

$$\begin{array}{l} V_{U}^{N}(W)=\frac{\omega_{U}\theta }{\rho +\delta }W+\frac{\omega_{E}\omega_{U}k_{E}{\left[\lambda_{E}(\rho +\delta )+\theta \eta_{E}\right]}^{2}}{(\mu_{E}k_{E}+2)\rho {\left(\rho +\delta \right)}^{2}}+\frac{\omega_{U}^{2}k_{U}{\left[\lambda_{U}(\rho +\delta )+\theta \eta_{U}\right]}^{2}}{2(\mu_{U}k_{U}+2)\rho {\left(\rho +\delta \right)}^{2}}+\\ \frac{\omega_{U}(1-\omega_{E}-\omega_{U}){\left[\lambda_{G}(\rho +\delta )+\theta \eta_{G}\right]}^{2}}{(\mu_{G}+\nu_{G})\rho {\left(\rho +\delta \right)}^{2}} \end{array}$$ (23)

$$\begin{array}{l} V_{G}^{N}(W)=\frac{(1-\omega_{E}-\omega_{U})\theta }{\rho +\delta }W+\frac{\omega_{E}(1-\omega_{E}-\omega_{U})k_{E}{\left[\lambda_{E}(\rho +\delta )+\theta \eta_{E}\right]}^{2}}{(\mu_{E}k_{E}+2)\rho {\left(\rho +\delta \right)}^{2}}+\\ \frac{\omega_{U}(1-\omega_{E}-\omega_{U})k_{U}{\left[\lambda_{U}(\rho +\delta )+\theta \eta_{U}\right]}^{2}}{(\mu_{U}k_{U}+2)\rho {\left(\rho +\delta \right)}^{2}}+\frac{{\left(1-\omega_{E}-\omega_{U}\right)}^{2}{\left[\lambda_{G}(\rho +\delta )+\theta \eta_{G}\right]}^{2}}{2(\mu_{G}+\nu_{G})\rho {\left(\rho +\delta \right)}^{2}} \end{array}$$ (24)

The optimal benefit function *V*(*W*) = *V*_*E*_(*W*)+*V*_*U*_(*W*)+*V*_*G*_(*W*) for the higher education ecosystem system can then be derived from *V*^*N*^(*W*) as follows:

$$\begin{array}{l} V^{N}(W)=\frac{\theta }{\rho +\delta }W+\frac{\omega_{E}(2-\omega_{E})k_{E}{\left[\lambda_{E}(\rho +\delta )+\theta \eta_{E}\right]}^{2}}{2(\mu_{E}k_{E}+2)\rho {\left(\rho +\delta \right)}^{2}}+\frac{\omega_{U}(2-\omega_{U})k_{U}{\left[\lambda_{U}(\rho +\delta )+\theta \eta_{U}\right]}^{2}}{2(\mu_{U}k_{U}+2)\rho {\left(\rho +\delta \right)}^{2}}+\\ \frac{{\left[1-(\omega_{E}+\omega_{U})\right]}^{2}{\left[\lambda_{G}(\rho +\delta )+\theta \eta_{G}\right]}^{2}}{2(\mu_{G}+\nu_{G})\rho {\left(\rho +\delta \right)}^{2}} \end{array}$$ (25)

### Cost-sharing model

Under the cost-sharing model, the government, as the leading party of the higher education ecosystem, shares some of the research and development costs between enterprises and universities through resource sharing and research and development subsidies to encourage enterprises and universities to invest more in research and development to enhance the overall innovation level of the ecosystem. The government grants cost subsidies to enterprises and universities are *α* and *β* (0≤*α*,*β*≤1), respectively.

To obtain the equilibrium strategy of the problem, the HJB equation is used to solve the problem according to the sufficient conditions of the static feedback Stackelberg equilibrium strategy. The innovation return functions of enterprises and universities are *V*_*E*_(*W*) and *V*_*U*_(*W*), respectively, which satisfy the following HJB equation:

$$\rho V_{E}(W)=\underset{E_{E}\geq 0}{\max}\{\omega_{E}\pi (t)+(\alpha -1)(\frac{\mu_{E}}{2}+\frac{1}{k_{E}})E_{E}^{2}+V_{E}^{'}(W)(\eta_{E}E_{E}+\eta_{U}E_{U}+\eta_{G}E_{G}-\delta W)\}$$ (26)

$$\rho V_{U}(W)=\underset{E_{U}\geq 0}{\max}\{\omega_{U}\pi (t)+(\beta -1)(\frac{\mu_{U}}{2}+\frac{1}{k_{U}})E_{U}^{2}+V_{U}^{'}(W)(\eta_{E}E_{E}+\eta_{U}E_{U}+\eta_{G}E_{G}-\delta W)\}$$ (27)

Taking the first-order partial derivatives of the right side of Eqs (26)–(27) with respect to *E*_*E*_ and *E*_*U*_ and setting them equal to zero, we obtain:

$$E_{E}=\frac{k_{E}[\omega_{E}\lambda_{E}+\eta_{E}V_{E}^{'}(W)]}{\left(1-\alpha )(\mu_{E}k_{E}+2\right)},\;E_{U}=\frac{k_{U}[\omega_{U}\lambda_{U}+\eta_{U}V_{U}^{'}(W)]}{\left(1-\beta )(\mu_{U}k_{U}+2\right)}$$ (28)

The government determines the level of its efforts and the proportion of cost subsidies to other subjects according to the decisions of enterprises and universities, and its HJB equation can be expressed as follows:

$$\begin{array}{l} \rho V_{G}(W)=\underset{E_{G}\geq 0}{\max}\{(1-\omega_{E}-\omega_{U})\pi (t)-\frac{1}{2}(\mu_{G}+\nu_{G})E_{G}^{2}(t)-\alpha (\frac{\mu_{E}}{2}+\frac{1}{k_{E}})E_{E}^{2}(t)-\\ \beta (\frac{\mu_{U}}{2}+\frac{1}{k_{U}})E_{U}^{2}(t)+V_{G}^{'}(W)(\eta_{E}E_{E}+\eta_{U}E_{U}+\eta_{G}E_{G}-\delta W)\} \end{array}$$ (29)

Substituting Eq (28) into Eq (29) and solving for the right end part to maximize the condition of Eq (29), the first-order partial derivatives are taken for *E*_*G*_, *α*, and *β* which is equal to zero to obtain:

$$E_{G}=\frac{\left[(1-\omega_{E}-\omega_{U})\lambda_{G}+\eta_{G}V_{G}^{'}(W)\right]}{\mu_{G}+\nu_{G}}$$ (30)

$$\alpha =\frac{\lambda_{E}(2-3\omega_{E}-2\omega_{U})+\eta_{E}[2V_{G}^{'}(W)-V_{E}^{'}(W)]}{\lambda_{E}(2-\omega_{E}-2\omega_{U})+\eta_{E}[2V_{G}^{'}(W)+V_{E}^{'}(W)]}$$ (31)

$$\beta =\frac{\lambda_{U}(2-3\omega_{E}-2\omega_{U})+\eta_{U}[2V_{G}^{'}(W)-V_{U}^{'}(W)]}{\lambda_{U}(2-2\omega_{E}-\omega_{U})+\eta_{U}[2V_{G}^{'}(W)+V_{U}^{'}(W)]}$$ (32)

Substituting the HJB equations for firms, universities, and government gives:

$$\begin{array}{l} \rho V_{E}(W)=(\omega_{E}\theta -\delta V_{E}^{'}(W))W+\frac{k_{E}(\omega_{E}\lambda_{E}+\eta_{E}V_{E}^{'}(W))[\lambda_{E}(2-\omega_{E}-2\omega_{U})+\eta_{E}(2V_{G}^{'}(W)+V_{E}^{'}(W))]}{4(\mu_{E}k_{E}+2)}+\\ \frac{k_{U}(\omega_{E}\lambda_{U}+\eta_{U}V_{E}^{'}(W))[\lambda_{U}(2-\omega_{U}-2\omega_{E})+\eta_{U}(2V_{G}^{'}(W)+V_{U}^{'}(W))]}{2(\mu_{U}k_{U}+2)}+\\ \frac{\left(\omega_{E}\lambda_{G}+\eta_{G}V_{E}^{'}(W))[\lambda_{G}(1-\omega_{E}-\omega_{U})+\eta_{G}V_{G}^{'}(W)\right]}{\mu_{G}+\nu_{G}} \end{array}$$ (33)

$$\begin{array}{l} \rho V_{U}(W)=(\omega_{U}\theta -\delta V_{U}^{'}(W))W+\frac{k_{E}(\omega_{U}\lambda_{E}+\eta_{E}V_{U}^{'}(W))[\lambda_{E}(2-\omega_{E}-2\omega_{U})+\eta_{E}(2V_{G}^{'}(W)+V_{E}^{'}(W))]}{2(\mu_{E}k_{E}+2)}+\\ \frac{k_{U}(\omega_{U}\lambda_{U}+\eta_{U}V_{U}^{'}(W))[\lambda_{U}(2-\omega_{U}-2\omega_{E})+\eta_{U}(2V_{G}^{'}(W)+V_{U}^{'}(W))]}{4(\mu_{U}k_{U}+2)}+\\ \frac{\left(\omega_{E}\lambda_{G}+\eta_{G}V_{U}^{'}(W))[\lambda_{G}(1-\omega_{E}-\omega_{U})+\eta_{G}V_{G}^{'}(W)\right]}{\mu_{G}+\nu_{G}} \end{array}$$ (34)

$$\begin{array}{l} \rho V_{G}(W)=[(1-\omega_{E}-\omega_{U})\theta -\delta V_{G}^{'}(W)]W+\frac{k_{E}{\left[\lambda_{E}(2-\omega_{E}-2\omega_{U})+\eta_{E}(2V_{G}^{'}(W)+V_{E}^{'}(W))\right]}^{2}}{8(\mu_{E}k_{E}+2)}+\\ \frac{k_{U}{\left[\lambda_{U}(2-\omega_{U}-2\omega_{E})+\eta_{U}(2V_{G}^{'}(W)+V_{U}^{'}(W))\right]}^{2}}{8(\mu_{U}k_{U}+2)}+\frac{{\left[\lambda_{G}(1-\omega_{E}-\omega_{U})+\eta_{G}V_{G}^{'}(W)\right]}^{2}}{2(\mu_{G}+\nu_{G})} \end{array}$$ (35)

According to Eq (33)—Eq (35), the linear optimal functions *V*_*E*_(*W*) = *f*_1_*W*+*f*_2_,*V*_*U*_(*W*) = *h*_1_*W*+*h*_2_, and *V*_*G*_(*W*) = *k*_1_*W*+*k*_2_ satisfy the above equation, where *f*_1_, *f*_2_, *h*_1_, *h*_2_, *k*_1_, and *k*_2_ are constants. Substituting *V*_*E*_(*W*), *V*_*U*_(*W*), *V*_*G*_(*W*) and their first-order derivatives with respect to *W*, we obtain:

$$\begin{array}{l} \rho (f_{1}W+f_{2})=(\omega_{E}\theta -\delta f_{1})W+\frac{k_{E}(\omega_{E}\lambda_{E}+\eta_{E}f_{1})[\lambda_{E}(2-\omega_{E}-2\omega_{U})+\eta_{E}(2k_{1}+f_{1})]}{4(\mu_{E}k_{E}+2)}+\\ \frac{k_{U}(\omega_{E}\lambda_{U}+\eta_{U}f_{1})[\lambda_{U}(2-\omega_{U}-2\omega_{E})+\eta_{U}(2k_{1}+h_{1})]}{2(\mu_{U}k_{U}+2)}+\frac{\left(\omega_{E}\lambda_{G}+\eta_{G}f_{1})[\lambda_{G}(1-\omega_{E}-\omega_{U})+\eta_{G}k_{1}\right]}{\mu_{G}+\nu_{G}} \end{array}$$ (36)

$$\begin{array}{l} \rho (h_{1}W+h_{2})=(\omega_{U}\theta -\delta h_{1})W+\frac{k_{E}(\omega_{U}\lambda_{E}+\eta_{E}h_{1})[\lambda_{E}(2-\omega_{E}-2\omega_{U})+\eta_{E}(2k_{1}+f_{1})]}{2(\mu_{E}k_{E}+2)}+\\ \frac{k_{U}(\omega_{U}\lambda_{U}+\eta_{U}h_{1})[\lambda_{U}(2-\omega_{U}-2\omega_{E})+\eta_{U}(2k_{1}+h_{1})]}{4(\mu_{U}k_{U}+2)}+\frac{\left(\omega_{E}\lambda_{G}+\eta_{G}h_{1})[\lambda_{G}(1-\omega_{E}-\omega_{U})+\eta_{G}k_{1}\right]}{\mu_{G}+\nu_{G}} \end{array}$$ (37)

$$\begin{array}{l} \rho (k_{1}W+k_{2})=[(1-\omega_{E}-\omega_{U})\theta -\delta k_{1}]W+\frac{k_{E}{\left[\lambda_{E}(2-\omega_{E}-2\omega_{U})+\eta_{E}(2k_{1}+f_{1})\right]}^{2}}{8(\mu_{E}k_{E}+2)}+\\ \frac{k_{U}{\left[\lambda_{U}(2-\omega_{U}-2\omega_{E})+\eta_{U}(2k_{1}+h_{1})\right]}^{2}}{8(\mu_{U}k_{U}+2)}+\frac{{\left[\lambda_{G}(1-\omega_{E}-\omega_{U})+\eta_{G}k_{1}\right]}^{2}}{2(\mu_{G}+\nu_{G})} \end{array}$$ (38)

The optimal return function can be found to have parameter values:

$$f_{1}^{S}=\frac{\omega_{E}\theta }{\rho +\delta },\;h_{1}^{S}=\frac{\omega_{U}\theta }{\rho +\delta },\;k_{1}^{S}=\frac{(1-\omega_{E}-\omega_{U})\theta }{\rho +\delta }$$ (39)

$$\begin{array}{l} f_{2}^{S}=\frac{\omega_{E}(2-\omega_{E}-2\omega_{U})k_{E}{\left[\lambda_{E}(\rho +\delta )+\theta \eta_{E}\right]}^{2}}{4(\mu_{E}k_{E}+2)\rho {\left(\rho +\delta \right)}^{2}}+\frac{\omega_{E}(2-\omega_{U}-2\omega_{E})k_{U}{\left[\lambda_{U}(\rho +\delta )+\theta \eta_{U}\right]}^{2}}{2(\mu_{U}k_{U}+2)\rho {\left(\rho +\delta \right)}^{2}}+\\ \frac{\omega_{E}(1-\omega_{E}-\omega_{U}){\left[\lambda_{G}(\rho +\delta )+\theta \eta_{G}\right]}^{2}}{(\mu_{G}+\nu_{G})\rho {\left(\rho +\delta \right)}^{2}} \end{array}$$ (40)

$$\begin{array}{l} h_{2}^{S}=\frac{\omega_{U}(2-\omega_{E}-2\omega_{U})k_{E}{\left[\lambda_{E}(\rho +\delta )+\theta \eta_{E}\right]}^{2}}{2(\mu_{E}k_{E}+2)\rho {\left(\rho +\delta \right)}^{2}}+\frac{\omega_{U}(2-\omega_{U}-2\omega_{E})k_{U}{\left[\lambda_{U}(\rho +\delta )+\theta \eta_{U}\right]}^{2}}{4(\mu_{U}k_{U}+2)\rho {\left(\rho +\delta \right)}^{2}}+\\ \frac{{\left(1-\omega_{E}-\omega_{U}\right)}^{2}{\left[\lambda_{G}(\rho +\delta )+\theta \eta_{G}\right]}^{2}}{(\mu_{G}+\nu_{G})\rho {\left(\rho +\delta \right)}^{2}} \end{array}$$ (41)

$$\begin{array}{l} k_{2}^{S}=\frac{{\left(2-\omega_{E}-2\omega_{U}\right)}^{2}k_{E}{\left[\lambda_{E}(\rho +\delta )+\theta \eta_{E}\right]}^{2}}{8(\mu_{E}k_{E}+2)\rho {\left(\rho +\delta \right)}^{2}}+\frac{{\left(2-\omega_{U}-2\omega_{E}\right)}^{2}k_{U}{\left[\lambda_{U}(\rho +\delta )+\theta \eta_{U}\right]}^{2}}{8(\mu_{U}k_{U}+2)\rho {\left(\rho +\delta \right)}^{2}}+\\ \frac{{\left(1-\omega_{E}-\omega_{U}\right)}^{2}{\left[\lambda_{G}(\rho +\delta )+\theta \eta_{G}\right]}^{2}}{2(\mu_{G}+\nu_{G})\rho {\left(\rho +\delta \right)}^{2}} \end{array}$$ (42)

Substituting $V_{E}^{'}(W)=f_{1}^{S}$, $V_{U}^{'}(W)=h_{1}^{S}$, and $V_{G}^{'}(W)=k_{1}^{S}$ into the HJB equation system, we obtain the optimal level of innovation input effort for each of the static feedback Nash equilibrium strategies of the enterprises, universities and the government under the cost-sharing model, as well as the subsidies given by the government to the enterprises and universities:

$$E_{E}^{S}=\frac{\left(2-\omega_{E}-2\omega_{U})k_{E}[\lambda_{E}(\rho +\delta )+\theta \eta_{E}\right]}{2(\mu_{E}k_{E}+2)(\rho +\delta )};$$

$$E_{U}^{S}=\frac{\left(2-\omega_{U}-2\omega_{E})k_{U}[\lambda_{U}(\rho +\delta )+\theta \eta_{U}\right]}{2(\mu_{U}k_{U}+2)(\rho +\delta )};$$

$$E_{G}^{S}=\frac{\left(1-\omega_{E}-\omega_{U})[\lambda_{G}(\rho +\delta )+\theta \eta_{G}\right]}{\left(\mu_{G}+\nu_{G})(\rho +\delta \right)}$$ (43)

$$\alpha =\{\begin{array}{l} \frac{\left(2-3\omega_{E}-2\omega_{U}\right)}{\left(2-\omega_{E}-2\omega_{U}\right)},3\omega_{E}+2\omega_{U}<2\\ 0,3\omega_{E}+2\omega_{U}\geq 2 \end{array}$$ (44)

$$\beta =\{\begin{array}{l} \frac{\left(2-3\omega_{U}-2\omega_{E}\right)}{\left(2-\omega_{U}-2\omega_{E}\right)},2\omega_{E}+3\omega_{U}<2\\ 0,2\omega_{E}+3\omega_{U}\geq 2 \end{array}$$ (45)

By substituting the solved parameter values into the optimal benefit functions of the enterprises, the universities and the government are collated:

$$\begin{array}{l} V_{E}^{S}(W)=\frac{\omega_{E}\theta }{\rho +\delta }W+\frac{\omega_{E}(2-\omega_{E}-2\omega_{U})k_{E}{\left[\lambda_{E}(\rho +\delta )+\theta \eta_{E}\right]}^{2}}{4(\mu_{E}k_{E}+2)\rho {\left(\rho +\delta \right)}^{2}}+\\ \frac{\omega_{E}(2-\omega_{U}-2\omega_{E})k_{U}{\left[\lambda_{U}(\rho +\delta )+\theta \eta_{U}\right]}^{2}}{2(\mu_{U}k_{U}+2)\rho {\left(\rho +\delta \right)}^{2}}+\frac{\omega_{E}(1-\omega_{E}-\omega_{U}){\left[\lambda_{G}(\rho +\delta )+\theta \eta_{G}\right]}^{2}}{(\mu_{G}+\nu_{G})\rho {\left(\rho +\delta \right)}^{2}} \end{array}$$ (46)

$$\begin{array}{l} V_{U}^{S}(W)=\frac{\omega_{U}\theta }{\rho +\delta }W+\frac{\omega_{U}(2-\omega_{E}-2\omega_{U})k_{E}{\left[\lambda_{E}(\rho +\delta )+\theta \eta_{E}\right]}^{2}}{2(\mu_{E}k_{E}+2)\rho {\left(\rho +\delta \right)}^{2}}+\\ \frac{\omega_{U}(2-\omega_{U}-2\omega_{E})k_{U}{\left[\lambda_{U}(\rho +\delta )+\theta \eta_{U}\right]}^{2}}{4(\mu_{U}k_{U}+2)\rho {\left(\rho +\delta \right)}^{2}}+\frac{\omega_{U}(1-\omega_{E}-\omega_{U}){\left[\lambda_{G}(\rho +\delta )+\theta \eta_{G}\right]}^{2}}{(\mu_{G}+\nu_{G})\rho {\left(\rho +\delta \right)}^{2}} \end{array}$$ (47)

$$\begin{array}{l} V_{G}^{S}(W)=\frac{(1-\omega_{E}-\omega_{U})\theta }{\rho +\delta }W+\frac{{\left(2-\omega_{E}-2\omega_{U}\right)}^{2}k_{E}{\left[\lambda_{E}(\rho +\delta )+\theta \eta_{E}\right]}^{2}}{8(\mu_{E}k_{E}+2)\rho {\left(\rho +\delta \right)}^{2}}+\\ \frac{{\left(2-\omega_{U}-2\omega_{E}\right)}^{2}k_{U}{\left[\lambda_{U}(\rho +\delta )+\theta \eta_{U}\right]}^{2}}{8(\mu_{U}k_{U}+2)\rho {\left(\rho +\delta \right)}^{2}}+\frac{{\left(1-\omega_{E}-\omega_{U}\right)}^{2}{\left[\lambda_{G}(\rho +\delta )+\theta \eta_{G}\right]}^{2}}{2(\mu_{G}+\nu_{G})\rho {\left(\rho +\delta \right)}^{2}} \end{array}$$ (48)

In turn, the optimal benefit function *V*^*S*^(*W*) for the higher education ecosystem system can be obtained from *V*(*W*) = *V*_*E*_(*W*)+*V*_*U*_(*W*)+*V*_*G*_(*W*):

$$\begin{array}{l} V^{S}(W)=\frac{\theta }{\rho +\delta }W+\frac{{\left[4-(\omega_{E}+2\omega_{U})\right]}^{2}k_{E}{\left[\lambda_{E}(\rho +\delta )+\theta \eta_{E}\right]}^{2}}{8(\mu_{E}k_{E}+2)\rho {\left(\rho +\delta \right)}^{2}}+\\ \frac{{\left[4-(2\omega_{E}+\omega_{U})\right]}^{2}k_{U}{\left[\lambda_{U}(\rho +\delta )+\theta \eta_{U}\right]}^{2}}{8(\mu_{U}k_{U}+2)\rho {\left(\rho +\delta \right)}^{2}}+\frac{{\left[1-(\omega_{E}+\omega_{U})\right]}^{2}{\left[\lambda_{G}(\rho +\delta )+\theta \eta_{G}\right]}^{2}}{2(\mu_{G}+\nu_{G})\rho {\left(\rho +\delta \right)}^{2}} \end{array}$$ (49)

### Collaborative research and development model

Under the collaborative R&D model, enterprises, universities and the government achieve in-depth synergy of strategies, organizations, resources and systems, constituting a unified system of interests, negotiating their optimal R&D strategies to maximize the overall benefits of the higher education ecosystem, and improving the overall level of innovation.

At this point, the objective function of the higher education ecosystem is:

$$\underset{E_{E};E_{U};E_{G}}{\max}J=J_{E}+J_{U}+J_{G}=\int_{0}^{\infty }e^{-\rho t}[\pi (t)-(\frac{\mu_{E}}{2}+\frac{1}{k_{E}})E_{E}^{2}(t)-(\frac{\mu_{U}}{2}+\frac{1}{k_{U}})E_{U}^{2}(t)-\frac{1}{2}(\mu_{G}+\nu_{G})E_{G}^{2}(t)]dt$$ (50)

To determine the equilibrium strategy for this problem, the HJB equation is used. The innovation benefit function of the higher education ecosystem is *V*(*W*), which satisfies the following HJB equation:

$$\begin{array}{l} \rho V(W)=\underset{E_{E};E_{U};E_{G}}{\max}\{\pi (t)-(\frac{\mu_{E}}{2}+\frac{1}{k_{E}})E_{E}^{2}(t)-(\frac{\mu_{U}}{2}+\frac{1}{k_{U}})E_{U}^{2}(t)-\frac{1}{2}(\mu_{G}+\nu_{G})E_{G}^{2}(t)+\\ V^{'}(W)(\eta_{E}E_{E}+\eta_{U}E_{U}+\eta_{G}E_{G}-\delta W)\} \end{array}$$ (51)

The right side of Eq (51) is obtained by taking the first-order partial derivatives of Eq (51) with respect to *E*_*E*_, *E*_*U*_, and *E*_*G*_ and setting them equal to zero:

$$E_{E}=\frac{k_{E}[\lambda_{E}+\eta_{E}V^{'}(W)]}{\mu_{E}k_{E}+2},\;E_{U}=\frac{k_{U}[\lambda_{U}+\eta_{U}V^{'}(W)]}{\mu_{U}k_{U}+2},\;E_{G}=\frac{\lambda_{G}+\eta_{G}V^{'}(W)}{\mu_{G}+\nu_{G}}$$ (52)

Substituting Eq (52) into Eq (51) gives:

$$\rho V(W)=[\theta -\delta V^{'}(W)]W+\frac{k_{E}{\left[\lambda_{E}+\eta_{E}V^{'}(W)\right]}^{2}}{2(\mu_{E}k_{E}+2)}+\frac{k_{U}{\left[\lambda_{U}+\eta_{U}V^{'}(W)\right]}^{2}}{2(\mu_{U}k_{U}+2)}+\frac{{\left[\lambda_{G}+\eta_{G}V^{'}(W)\right]}^{2}}{2(\mu_{G}+\nu_{G})}$$ (53)

According to Eq (53), the linear optimal function *V*(*W*) = *f*_1_*W*+*f*_2_ satisfies the above equation, where *f*_1_ and *f*_2_ are constants. Substituting *V*(*W*) and its first-order derivative with respect to *W*, we obtain:

$$\rho V(f_{1}W+f_{2})=(\theta -\delta f_{1})W+\frac{k_{E}{\left(\lambda_{E}+\eta_{E}f_{1}\right)}^{2}}{2(\mu_{E}k_{E}+2)}+\frac{k_{U}{\left(\lambda_{U}+\eta_{U}f_{1}\right)}^{2}}{2(\mu_{U}k_{U}+2)}+\frac{{\left(\lambda_{G}+\eta_{G}f_{1}\right)}^{2}}{2(\mu_{G}+\nu_{G})}$$ (54)

The parameter values of the optimal return function can be found as:

$$f_{1}^{C}=\frac{\theta }{\rho +\delta }$$ (55)

$$f_{2}^{C}=\frac{k_{E}{\left[\lambda_{E}(\rho +\delta )+\theta \eta_{E}\right]}^{2}}{2(\mu_{E}k_{E}+2)\rho {\left(\rho +\delta \right)}^{2}}+\frac{k_{U}{\left[\lambda_{U}(\rho +\delta )+\theta \eta_{U}\right]}^{2}}{2(\mu_{U}k_{U}+2)\rho {\left(\rho +\delta \right)}^{2}}+\frac{{\left[\lambda_{G}(\rho +\delta )+\theta \eta_{G}\right]}^{2}}{2(\mu_{G}+\nu_{G})\rho {\left(\rho +\delta \right)}^{2}}$$ (56)

Substituting $V^{'}(W)=f_{1}^{C}$ into the system of HJB equations, the collation yields the optimal level of innovation input effort for each of the static feedback Nash equilibrium strategies of enterprises, universities and the government under the collaborative, cooperative research and development model as follows:

$$E_{E}^{C}=\frac{k_{E}[\lambda_{E}(\rho +\delta )+\theta \eta_{E}]}{\left(\mu_{E}k_{E}+2)(\rho +\delta \right)},\;E_{U}^{C}=\frac{k_{U}[\lambda_{U}(\rho +\delta )+\theta \eta_{U}]}{\left(\mu_{U}k_{U}+2)(\rho +\delta \right)},\;E_{G}^{C}=\frac{\left[\lambda_{G}(\rho +\delta )+\theta \eta_{G}\right]}{\left(\mu_{G}+\nu_{G})(\rho +\delta \right)}$$ (57)

The solved parameter values are subsequently collated into the optimal benefit function for the higher education ecosystem:

$$V^{C}(W)=\frac{\theta }{\rho +\delta }W+\frac{k_{E}{\left[\lambda_{E}(\rho +\delta )+\theta \eta_{E}\right]}^{2}}{2(\mu_{E}k_{E}+2)\rho {\left(\rho +\delta \right)}^{2}}+\frac{k_{U}{\left[\lambda_{U}(\rho +\delta )+\theta \eta_{U}\right]}^{2}}{2(\mu_{U}k_{U}+2)\rho {\left(\rho +\delta \right)}^{2}}+\frac{{\left[\lambda_{G}(\rho +\delta )+\theta \eta_{G}\right]}^{2}}{2(\mu_{G}+\nu_{G})\rho {\left(\rho +\delta \right)}^{2}}$$ (58)

The optimal benefit functions for enterprises, universities and the government are as follows:

$$\begin{array}{l} V_{E}^{C}(W)=\frac{\omega_{E}\theta }{\rho +\delta }W+\frac{\omega_{E}k_{E}{\left[\lambda_{E}(\rho +\delta )+\theta \eta_{E}\right]}^{2}}{2(\mu_{E}k_{E}+2)\rho {\left(\rho +\delta \right)}^{2}}+\frac{\omega_{E}k_{U}{\left[\lambda_{U}(\rho +\delta )+\theta \eta_{U}\right]}^{2}}{2(\mu_{U}k_{U}+2)\rho {\left(\rho +\delta \right)}^{2}}+\\ \frac{\omega_{E}{\left[\lambda_{G}(\rho +\delta )+\theta \eta_{G}\right]}^{2}}{2(\mu_{G}+\nu_{G})\rho {\left(\rho +\delta \right)}^{2}} \end{array}$$ (59)

$$\begin{array}{l} V_{U}^{C}(W)=\frac{\omega_{U}\theta }{\rho +\delta }W+\frac{\omega_{U}k_{E}{\left[\lambda_{E}(\rho +\delta )+\theta \eta_{E}\right]}^{2}}{2(\mu_{E}k_{E}+2)\rho {\left(\rho +\delta \right)}^{2}}+\frac{\omega_{U}k_{U}{\left[\lambda_{U}(\rho +\delta )+\theta \eta_{U}\right]}^{2}}{2(\mu_{U}k_{U}+2)\rho {\left(\rho +\delta \right)}^{2}}+\\ \frac{\omega_{U}{\left[\lambda_{G}(\rho +\delta )+\theta \eta_{G}\right]}^{2}}{2(\mu_{G}+\nu_{G})\rho {\left(\rho +\delta \right)}^{2}} \end{array}$$ (60)

$$\begin{array}{l} V_{G}^{C}(W)=\frac{(1-\omega_{G}-\omega_{U})\theta }{\rho +\delta }W+\frac{(1-\omega_{G}-\omega_{U})k_{E}{\left[\lambda_{E}(\rho +\delta )+\theta \eta_{E}\right]}^{2}}{2(\mu_{E}k_{E}+2)\rho {\left(\rho +\delta \right)}^{2}}+\\ \frac{(1-\omega_{G}-\omega_{U})k_{U}{\left[\lambda_{U}(\rho +\delta )+\theta \eta_{U}\right]}^{2}}{2(\mu_{U}k_{U}+2)\rho {\left(\rho +\delta \right)}^{2}}+\frac{(1-\omega_{G}-\omega_{U}){\left[\lambda_{G}(\rho +\delta )+\theta \eta_{G}\right]}^{2}}{2(\mu_{G}+\nu_{G})\rho {\left(\rho +\delta \right)}^{2}} \end{array}$$ (61)

## Comparative analysis of balanced results

The following conclusions can be drawn by comparing the optimal level of innovation effort, the optimal innovation benefit, and the total benefit of the higher education ecosystem as a whole for enterprises, universities and the government under the above three R&D models.

**Proposition 1:** The government’s research and development effort is the same in both the noncollaborative research and development model and the cost-sharing model, but cost-sharing from the government can make the research and development effort of enterprises and universities increase significantly by an amount equal to the proportion of the government’s cost-sharing to the two, which suggests that cost-sharing as a kind of incentive mechanism can enhance the research and development effort of enterprises and universities; in the collaborative research and development model, the research and development effort of enterprises, universities and the government is better than that of the other two research and development models.

**Proof:** The formula above gives (where $0<\omega_{E}<\frac{2}{3}$, $0<\omega_{U}<\frac{2}{3}$):

$$\begin{array}{l} E_{E}^{S}-E_{E}^{N}=\frac{\left(2-3\omega_{E}-2\omega_{U})[\lambda_{E}(\rho +\delta )+\theta \eta_{E}\right]}{2(\mu_{E}k_{E}+2)(\rho +\delta )}=\\ \frac{\left(2-\omega_{E}-2\omega_{U})[\lambda_{E}(\rho +\delta )+\theta \eta_{E}\right]}{2(\mu_{E}k_{E}+2)(\rho +\delta )}\cdot \frac{2-3\omega_{E}-2\omega_{U}}{2-\omega_{E}-2\omega_{U}}=E_{E}^{S}\cdot \alpha >0 \end{array}$$ (62)

$$\begin{array}{l} E_{U}^{S}-E_{U}^{N}=\frac{\left(2-3\omega_{U}-2\omega_{E})[\lambda_{U}(\rho +\delta )+\theta \eta_{U}\right]}{2(\mu_{U}k_{U}+2)(\rho +\delta )}=\\ \frac{\left(2-\omega_{U}-2\omega_{E})[\lambda_{E}(\rho +\delta )+\theta \eta_{E}\right]}{2(\mu_{E}k_{E}+2)(\rho +\delta )}\cdot \frac{2-3\omega_{U}-2\omega_{E}}{2-\omega_{U}-2\omega_{E}}=E_{U}^{S}\cdot \beta >0 \end{array}$$ (63)

$$E_{G}^{N}=E_{G}^{S}$$ (64)

$$E_{E}^{C}-E_{E}^{S}=\frac{\left(\omega_{E}+2\omega_{U})[\lambda_{E}(\rho +\delta )+\theta \eta_{E}\right]}{2(\mu_{E}k_{E}+2)(\rho +\delta )}>0$$ (65)

$$E_{U}^{C}-E_{U}^{S}=\frac{\left(2\omega_{E}+\omega_{U})[\lambda_{U}(\rho +\delta )+\theta \eta_{U}\right]}{2(\mu_{U}k_{U}+2)(\rho +\delta )}>0$$ (66)

$$E_{G}^{C}-E_{G}^{S}=\frac{\left(\omega_{E}+\omega_{U})[\lambda_{G}(\rho +\delta )+\theta \eta_{G}\right]}{\left(\mu_{G}+\nu_{G})(\rho +\delta \right)}>0$$ (67)

### End of proof

**Proposition 2:** Under the enterprise cost-sharing model, the R&D returns of enterprises, universities and the government are all better than those under the noncooperative R&D model. When the government shares a portion of the R&D costs of enterprises and universities, the R&D returns of all three are enhanced, and a Pareto improvement is realized.

**Proof:** From the formula above (where $0<\omega_{E}<\frac{2}{3}$, $0<\omega_{U}<\frac{2}{3}$):

$$V_{E}^{S}-V_{E}^{N}=\frac{\omega_{E}(2-3\omega_{E}-2\omega_{U}){\left[\lambda_{E}(\rho +\delta )+\theta \eta_{E}\right]}^{2}}{4(\mu_{E}k_{E}+2)\rho {\left(\rho +\delta \right)}^{2}}+\frac{\omega_{E}(2-3\omega_{U}-2\omega_{E}){\left[\lambda_{U}(\rho +\delta )+\theta \eta_{U}\right]}^{2}}{2(\mu_{U}k_{U}+2)\rho {\left(\rho +\delta \right)}^{2}}>0$$ (68)

$$V_{U}^{S}-V_{U}^{N}=\frac{\omega_{U}(2-3\omega_{E}-2\omega_{U}){\left[\lambda_{E}(\rho +\delta )+\theta \eta_{E}\right]}^{2}}{2(\mu_{E}k_{E}+2)\rho {\left(\rho +\delta \right)}^{2}}+\frac{\omega_{U}(2-3\omega_{U}-2\omega_{E}){\left[\lambda_{U}(\rho +\delta )+\theta \eta_{U}\right]}^{2}}{4(\mu_{U}k_{U}+2)\rho {\left(\rho +\delta \right)}^{2}}>0$$ (69)

$$V_{G}^{S}-V_{G}^{N}=\frac{{\left(3\omega_{E}+2\omega_{U}-2\right)}^{2}{\left[\lambda_{E}(\rho +\delta )+\theta \eta_{E}\right]}^{2}}{8(\mu_{E}k_{E}+2)\rho {\left(\rho +\delta \right)}^{2}}+\frac{{\left(3\omega_{U}+2\omega_{E}-2\right)}^{2}{\left[\lambda_{U}(\rho +\delta )+\theta \eta_{U}\right]}^{2}}{8(\mu_{U}k_{U}+2)\rho {\left(\rho +\delta \right)}^{2}}>0$$ (70)

### End of proof

**Proposition 3:** The total benefits to the higher education ecosystem are greater in the collaborative research and development model than in the other two models, achieving Pareto optimality.

**Proof:** From the formula above (where $0<\omega_{E}<\frac{2}{3}$, $0<\omega_{U}<\frac{2}{3}$):

$$\begin{array}{l} V^{S}(W)-V^{N}(W)=\frac{(3\omega_{E}+2\omega_{U}-2)(\omega_{E}-2\omega_{U}-2){\left[\lambda_{E}(\rho +\delta )+\theta \eta_{E}\right]}^{2}}{8(\mu_{E}k_{E}+2)\rho {\left(\rho +\delta \right)}^{2}}+\\ \frac{(2\omega_{E}+3\omega_{U}-2)(-2\omega_{E}+\omega_{U}-2){\left[\lambda_{U}(\rho +\delta )+\theta \eta_{U}\right]}^{2}}{8(\mu_{U}k_{U}+2)\rho {\left(\rho +\delta \right)}^{2}}>0 \end{array}$$ (71)

$$\begin{array}{l} V^{C}(W)-V^{S}(W)=\frac{{\left(\omega_{E}+\omega_{U}\right)}^{2}{\left[\lambda_{E}(\rho +\delta )+\theta \eta_{E}\right]}^{2}}{2(\mu_{E}k_{E}+2)\rho {\left(\rho +\delta \right)}^{2}}+\\ \frac{{\left(\omega_{E}+2\omega_{U}\right)}^{2}{\left[\lambda_{U}(\rho +\delta )+\theta \eta_{U}\right]}^{2}}{8(\mu_{U}k_{U}+2)\rho {\left(\rho +\delta \right)}^{2}}+\frac{{\left(2\omega_{E}+\omega_{U}\right)}^{2}{\left[\lambda_{G}(\rho +\delta )+\theta \eta_{G}\right]}^{2}}{8(\mu_{G}+\nu_{G})\rho {\left(\rho +\delta \right)}^{2}}>0 \end{array}$$ (72)

### End of proof

Although the total benefits to the higher education ecosystem can be maximized under the collaborative research and development model, the constraints of Eq (73)–Eq (75) need to be met for enterprises, universities and the government to participate in the synergy voluntarily and for the benefits to each of the three parties under the collaborative research and development model to be greater than those under the other models.


$$\Delta V_{E}(W)=V_{E}^{C}-V_{E}^{S}\geq 0$$ (73)

$$\Delta V_{U}(W)=V_{U}^{C}-V_{U}^{S}\geq 0$$ (74)

$$\Delta V_{G}(W)=V_{G}^{C}-V_{G}^{S}\geq 0$$ (75)

In addition, the respective incremental shares of the total higher education ecosystem benefits for enterprises, universities, and the government should be negotiated based on the three parties’ bargaining power. At the same time, the binding terms and incentives for collaborative research and development in higher education ecosystems need to be gradually improved in practice.

**Proposition 4:** Government cost-sharing between enterprises and universities can contribute to the technological level of the higher education ecosystem. Under the collaborative R&D model, the technological level of the higher education ecosystem is at its highest, as enterprises and universities maximize their efforts in technological research and development and innovation.

**Proof:** It follows from Proposition 1 that:

$\frac{\eta_{E}E_{E}^{C}+\eta_{U}E_{U}^{C}+\eta_{G}E_{G}^{C}}{\delta }>\frac{\eta_{E}E_{E}^{S}+\eta_{U}E_{U}^{S}+\eta_{G}E_{G}^{S}}{\delta }>\frac{\eta_{E}E_{E}^{N}+\eta_{U}E_{U}^{N}+\eta_{G}E_{G}^{N}}{\delta }$, *ψ*^*c*^>*ψ*^*s*^>*ψ*^*N*^. Additionally, Eq (76) is shown.


$$\frac{dW}{d\psi }=1-e^{-\delta t}>0$$ (76)

*W* is an increasing function of *ψ*. Therefore, for *W*^*C*^(*t*)>*W*^*S*^(*t*)>*W*^*N*^(*t*), the proof is complete.

## Numerical simulation

### Comparison of the optimal returns of the three models

Based on the above model analysis, the optimal level of effort, the optimal benefit, and the optimal benefit of the innovation ecosystem as a whole of the three types of subjects in different R&D cooperation modes depend on the parameter assignment. Combining the logic of parameter assignment by scholars [29] and considering the actual situation of innovation balance in the higher education ecosystem in the context of digitalization, the parameter assignment in this paper is shown in Table 2.

**Table 2:** Parameters and assignments.

Parameter	Assignment	Parameter	Assignment	Parameter	Assignment
*μ*_*E*_	0.5	*k*_*U*_	0.2	*λ*_*U*_	0.8
*μ*_*U*_	0.4	*η*_*E*_	0.3	*λ*_*G*_	0.6
*μ*_*G*_	0.2	*η* _ *U* _	0.4	*θ*	0.4
*v*_*G*_	0.1	*η*_*G*_	0.2	*ρ*	0.1
*k*_*E*_	0.3	*λ*_*E*_	0.7	*δ*	0.1
*W*_0_	2	*ω*_*E*_	0.25	*ω*_*U*_	0.15

Based on the parameter assignments in Table 2, the equilibrium results of enterprises, universities, and governments in the higher education ecosystem under the three R&D modes were calculated, as shown in Table 3.

**Table 3:** Comparative analysis of equilibrium results of higher education ecosystems in the three models.

Parameter	Noncooperative research and development model	Cost-sharing model	Collaborative research and development model
*E*_*E*_	0.53	1.26	2.28
*E*_*U*_	0.90	1.43	2.65
*E*_*G*_	1.40	1.40	3.20
*α*	0	0.60	-
*β*	0	0.45	-
*W*	4.38‒2.43*e*_‒0.1*t*_	6.41‒4.23*e*^‒0.1*t*^	11.31‒6.67e^‒0.1*t*^
*V*_*E*_	0.2*W*+4.40	0.2*W*+6.37	-
*V*_*U*_	0.8*W*+3.34	0.8*W*+7.03	-
*V*_*G*_	*W*+6.78	*W*+9.12	-
*V*	2*W*+14.52	2*W*+22.52	2*W*+27.89

**Fig 2:**
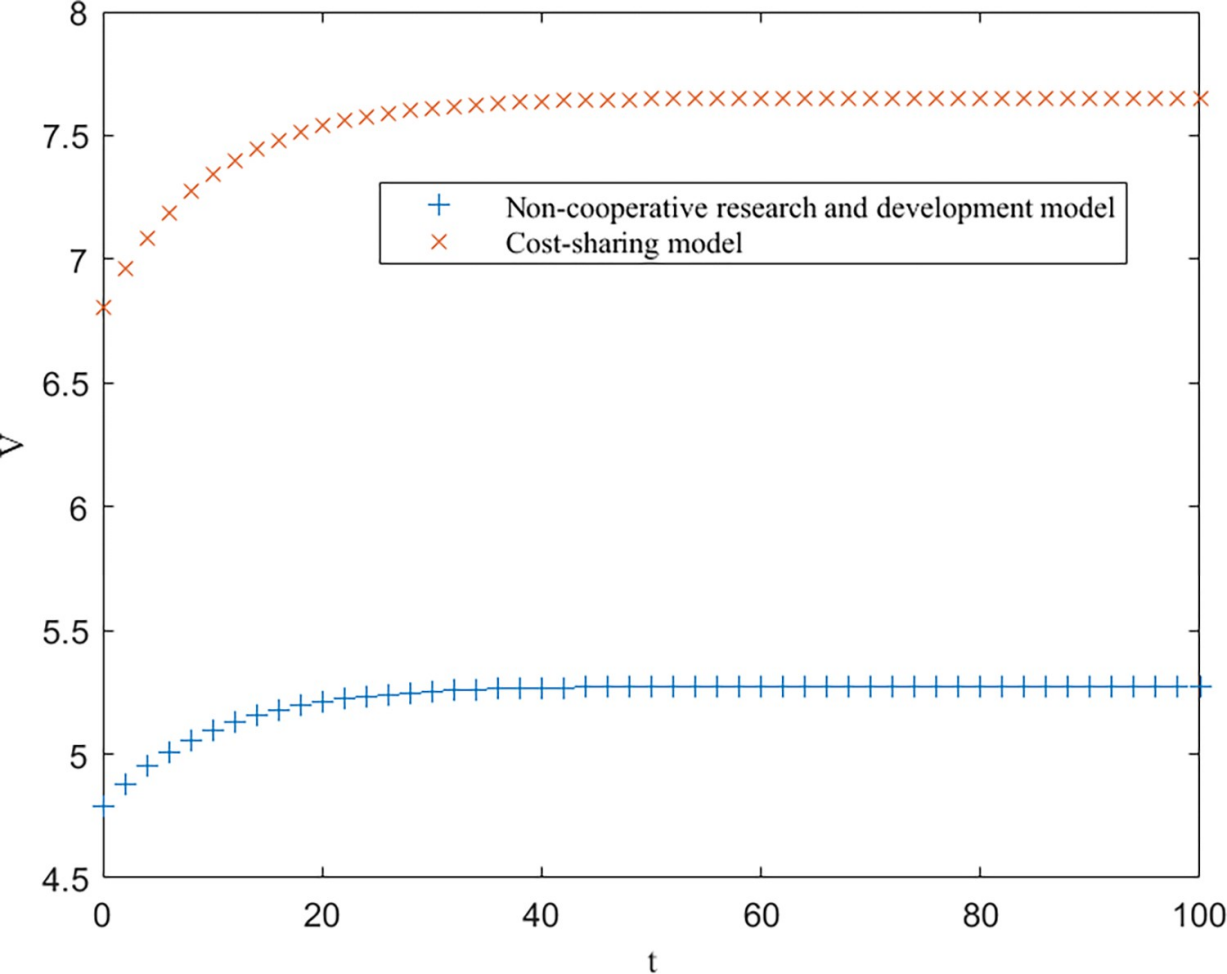
Optimal returns for the enterprises.

**Fig 3:**
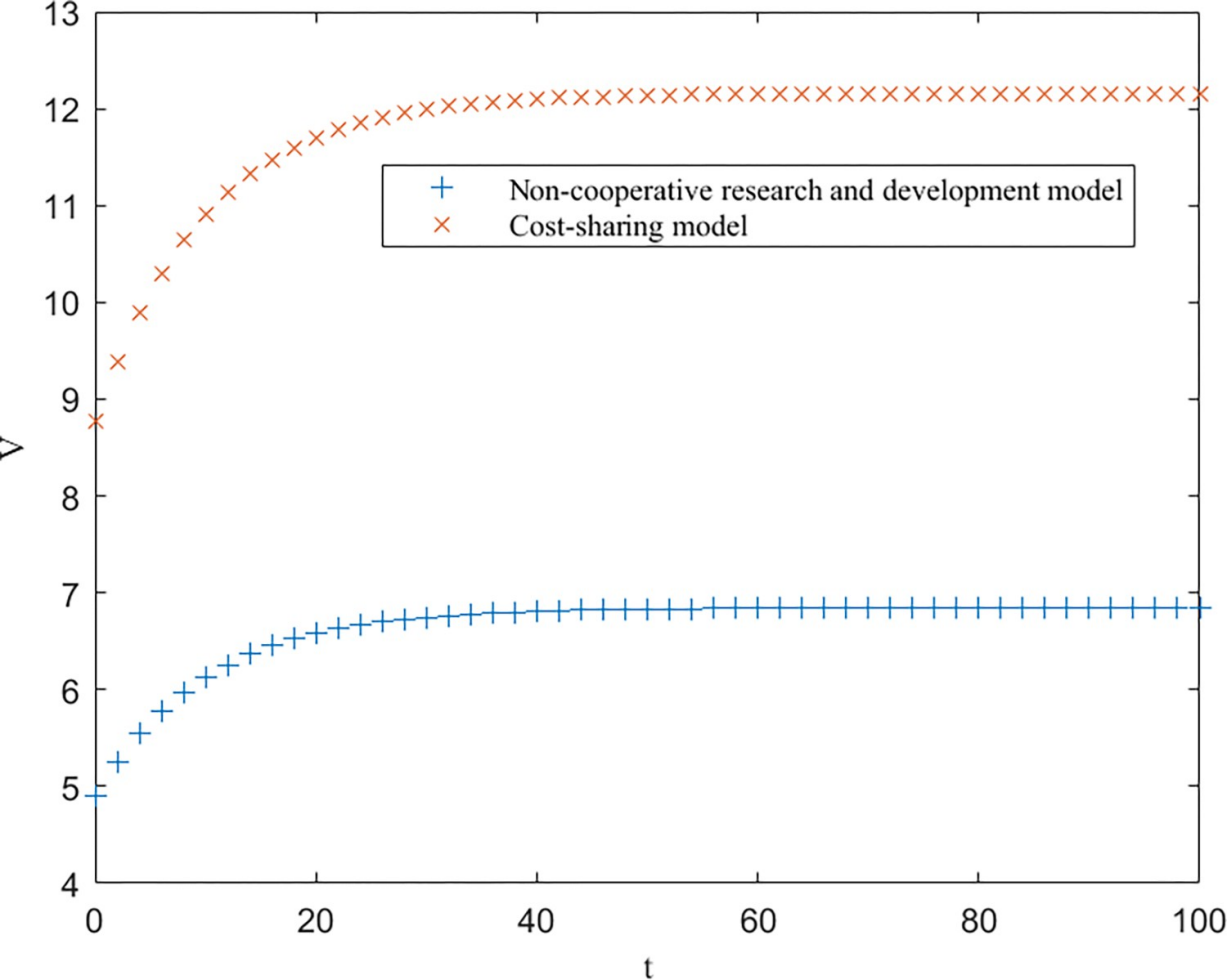
Optimal returns for universities.

**Fig 4:**
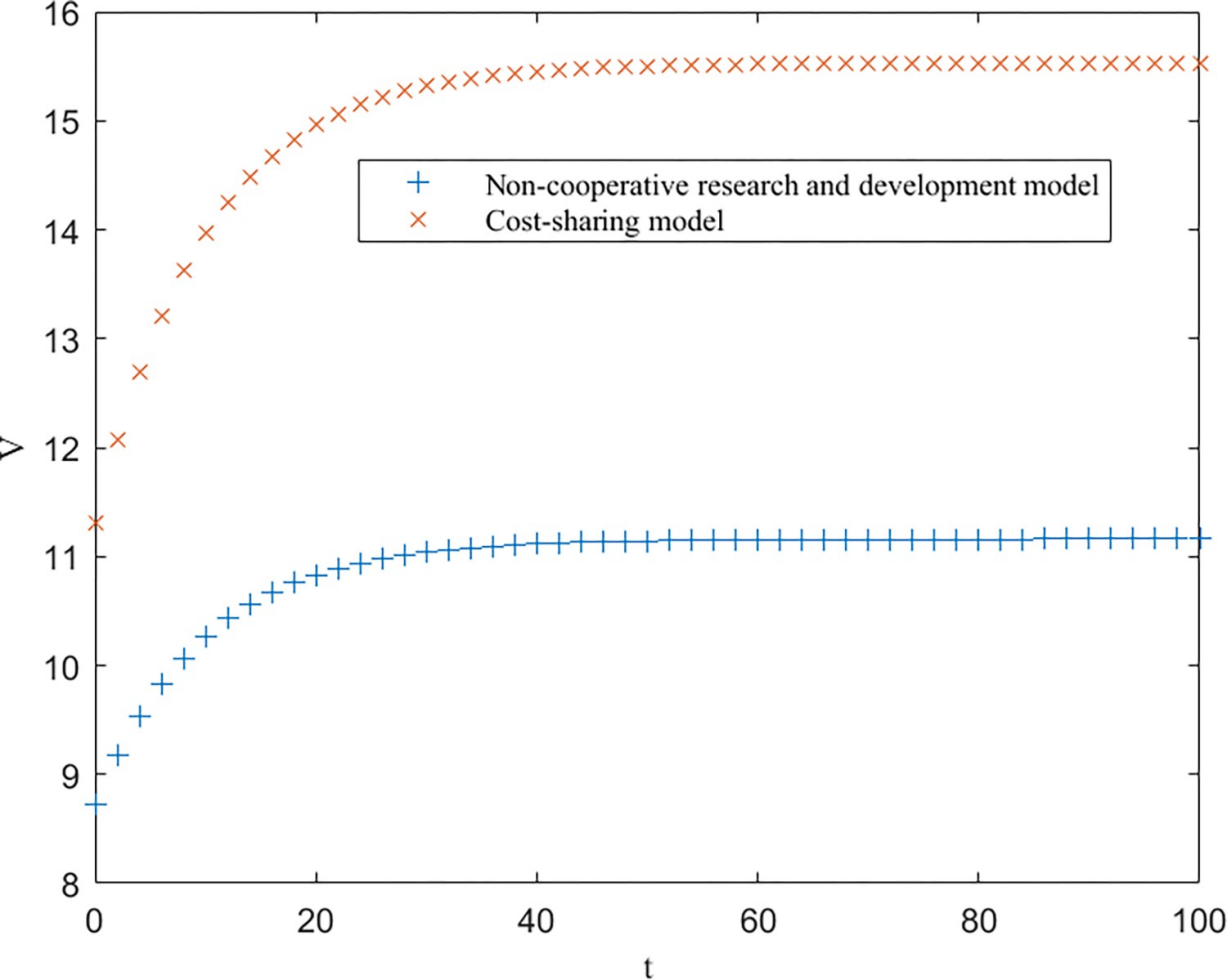
Optimal return for governments.

As shown in Table 3, $E_{E}^{N}<E_{E}^{S}<E_{E}^{C}$, $E_{U}^{N}<E_{U}^{S}<E_{U}^{C}$, and $E_{G}^{N}=E_{G}^{S}<E_{G}^{C}$ are consistent with the conclusions of Proposition 1. Furthermore, based on Table 3, the trends of the optimal returns of each of the three modes of enterprises, universities and government over time, as well as the trajectories of the optimal returns and the level of key core technologies of the higher education ecosystem as a whole, are plotted, as shown in Figs 2–4.

From Figs 2–4, it can be seen that the optimal benefit levels of enterprises, universities and the government first increase rapidly and then level off and converge to the equilibrium state with increasing time. The optimal benefit level of each party in the government cost-sharing model is always greater than that in the non-cooperative research and development mode, indicating that the Pareto improvement of the benefits of each party involved in the higher education ecosystem can be achieved through the cost-sharing mechanism, which is consistent with the conclusion of Proposition 2. Among the three modes of cooperation, the collaborative R&D mode is the optimal path, in which the benefits of all the participating subjects and the higher education ecosystem are greatly enhanced. This is because the government plays a stronger role in this model, effectively mobilizes the enthusiasm of all parties involved, and the sharing and utilization of resources by all parties is the highest, which directly improves the collaboration efficiency and innovation level of the higher education ecosystem.

**Fig 5:**
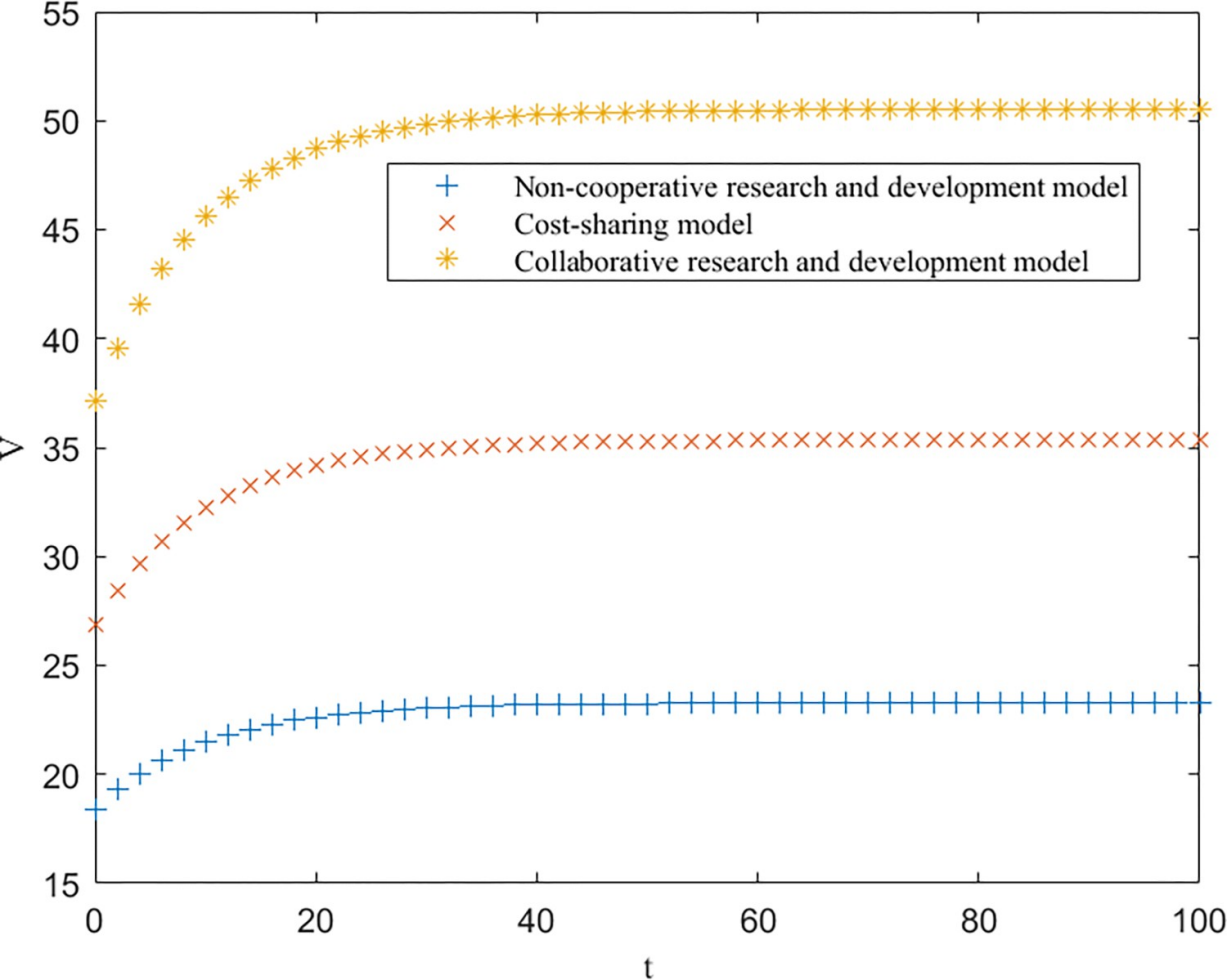
Comparison of overall higher education ecosystem benefits under the three models.

Fig 5 depicts the comparative relationships among the overall benefits of the higher education ecosystem under the noncooperative R&D mode, the cost-sharing mode and the collaborative R&D mode. The effect of improving the benefits of all parties in the higher education ecosystem through the cost-sharing mechanism is not as significant as that under the collaborative research and development mode, and the overall benefits of the higher education ecosystem have reached the optimal level under the collaborative R&D mode. This shows that collaborative research and development benefits in higher education ecosystems should be redistributed well. Under the three models, the relationship of innovation subjects changes from decentralized to centralized and then to modularized, the degree of connection gradually increases, the sharing of resources and information reaches the highest level, and there is a trend of iterative upgrading to a higher level. In addition, the technological maturity of innovators under the collaborative R&D model also reaches a high level, effectively promoting the technological progress and development of the higher education ecosystem and thus generating the desired benefits. This is consistent with the conclusion of Proposition 3.

**Fig 6:**
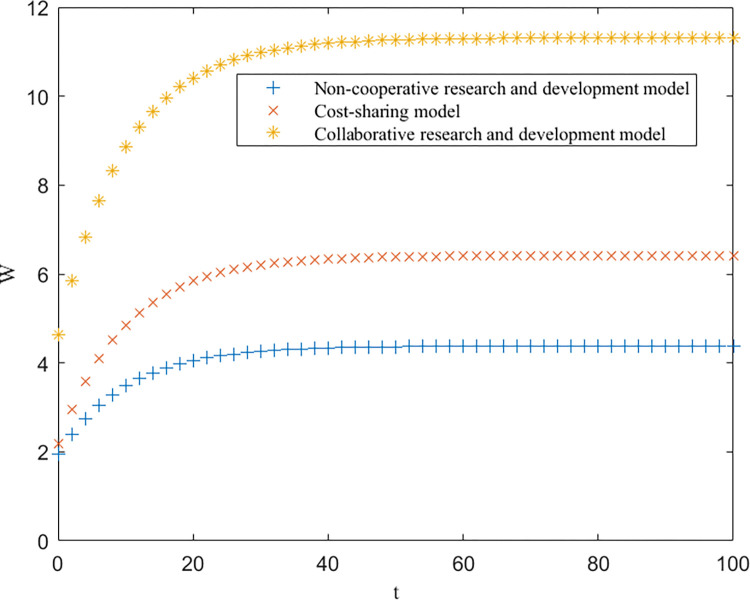
Comparison of technology levels in higher education ecosystems under three models.

Fig 6 depicts the comparative relationship between the technological level of the higher education ecosystem under the three R&D modes. The technological level of the higher education ecosystem increases rapidly with increasing time and then tends to reach a steady state, which indicates that the process of collaborative research and development in the higher education ecosystem is adjustable. A comprehensive comparison of the three R&D models reveals that the collaborative R&D model is the best, the cost-sharing model is the second best, and the noncollaborative R&D model is the worst, reflecting that the collaborative mechanism produces a multiplier effect; that is, as the government’s efforts become stronger and the technological maturity of enterprises and universities increases, the rate of technological upgrading of higher education ecosystems becomes faster. This also verifies the conclusion of Proposition 4.

### Impact of important parameters in the synergy model

**Fig 7:**
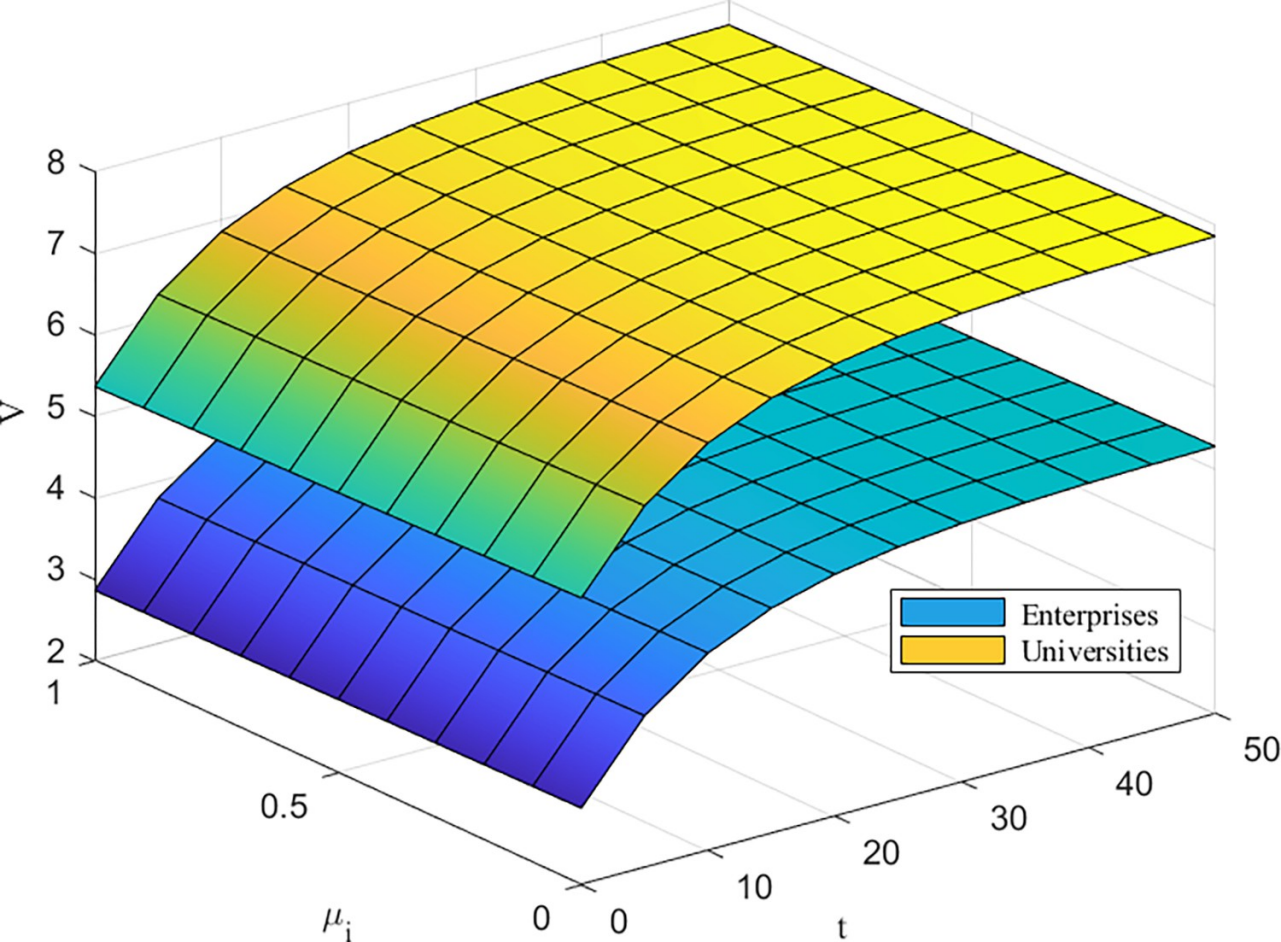
*μ*_*E*_, *μ*_*U*_ impact on*V*^*C*^(*W*).

**Fig 8:**
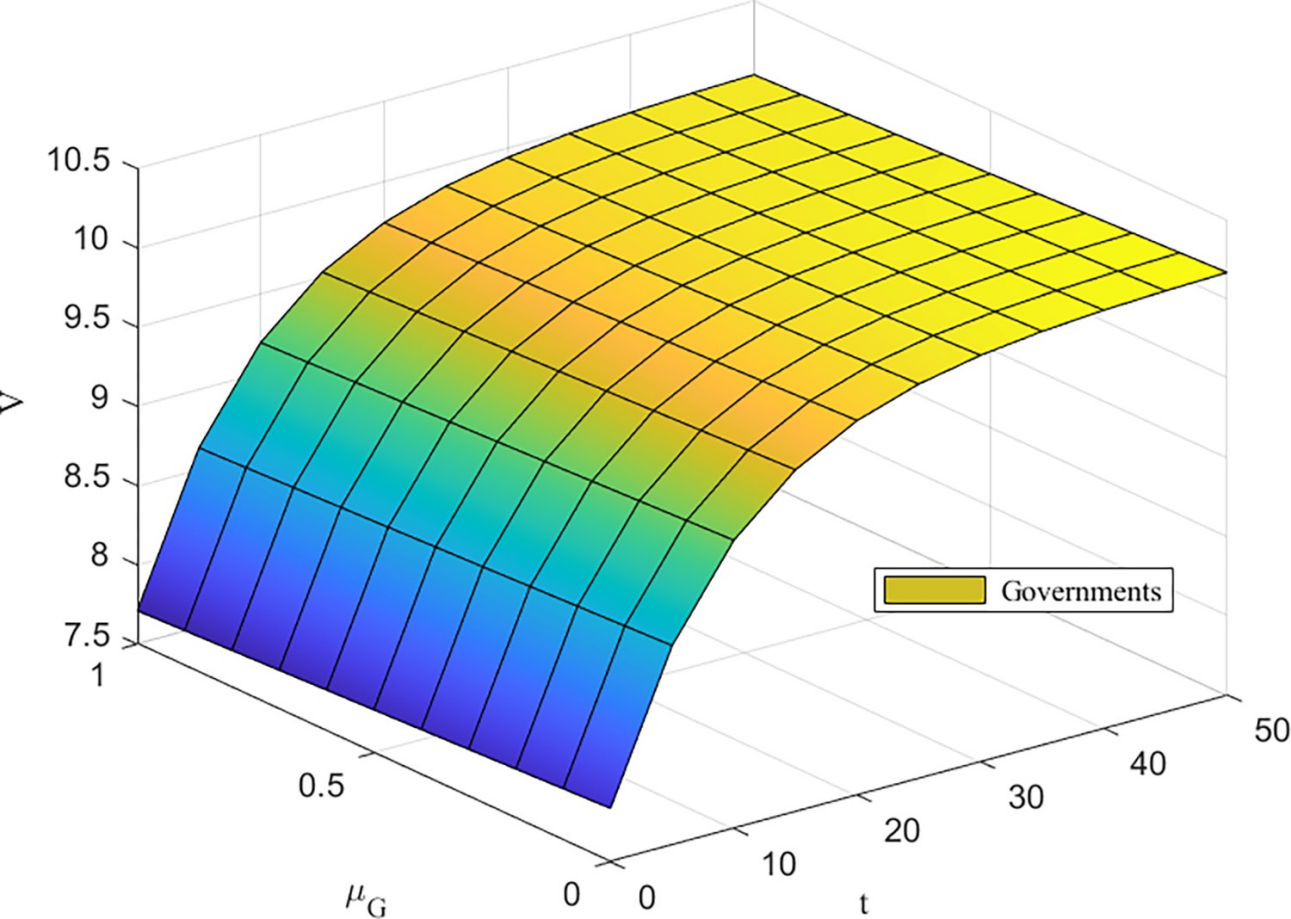
*μ_G_* impacts on *V*^*C*^(*W*).

Figs 7 and 8 depict the impact of the cost-of-effort coefficient on returns to the higher education ecosystem. The impact of the cost of effort on returns is small for enterprises and universities, while the impact of the cost of effort on returns is largest for governments and deepens over time. When *μ*_*G*_ is small, the negative impact of *μ*_*G*_ on earnings is small in the early part of cooperation and decreases rapidly as *μ*_*G*_ increases in the later part of cooperation. It is worth noting that the government’s actions play important macroregulatory and supervisory roles in the higher education innovation ecosystem, and the greater its degree of involvement is, the more likely it is to change the trend of the system’s benefits, which means that government involvement has an important impact on the higher education innovation ecosystem.

**Fig 9:**
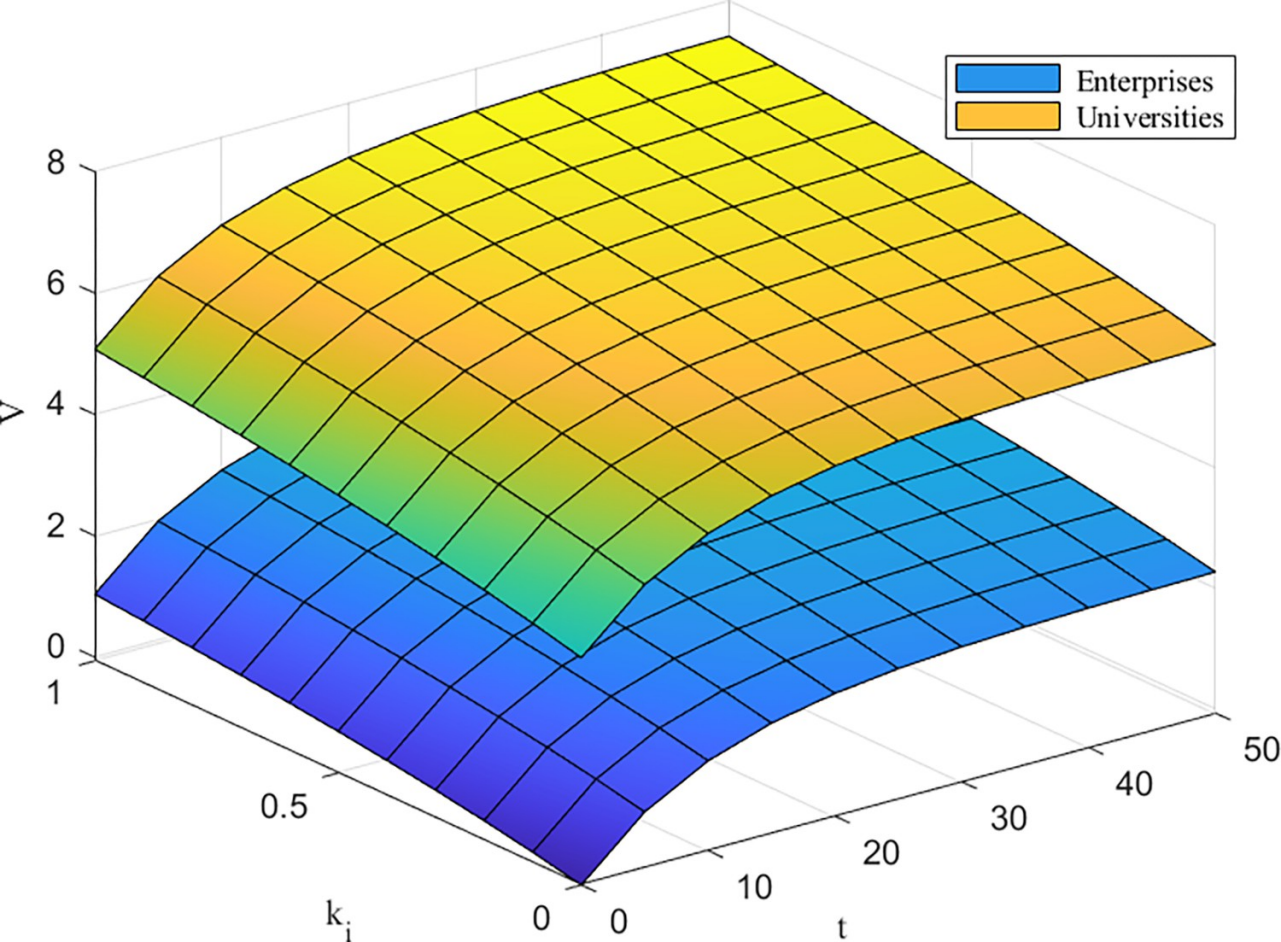
*k_i_* impacts on*V*^*C*^(*W*).

Fig 9 shows that as *k*^*i*^ increases, *V*^*C*^(*W*) gradually increases, and the benefits to HEIs are greater than those to firms. This matches the central position of universities in the higher education ecosystem, which increases their respective benefits and the overall benefits of the system by guiding firms to actively participate in the technological innovation process. On the one hand, the active participation of universities and companies changes the participation strategy, which in turn leads to a change in technology maturity; on the other hand, the change in technology maturity affects the level of revenue enhancement, which further affects the operational status of the system.

### Discussion of the simulation results

It can be found through numerical simulation that variables such as the benefits of the participating subjects, the benefits of the higher education ecosystem and technological maturity all reach the highest level under the collaborative R&D model and the lowest level under the noncollaborative R&D model, which reflects that the dynamic evolutionary path of the higher education ecosystem follows the noncollaborative R&D to the cost-sharing model and then the collaborative and centralized model. Different from the numerical simulation in previous studies, this paper not only considers the changes in the benefits and technology levels of the subjects under the three modes but also focuses on analyzing the impact of important variables under the collaborative mode, which helps the higher education ecosystem iteratively upgrade to a higher level of energy under the collaborative model. In addition, this paper innovatively introduces variables such as technology maturity, which also fills the gap in the numerical simulation of previous studies to a certain extent. simulation.

## Conclusions and implications

### Main conclusions

This paper focuses on the collaborative research and development strategy of the higher education ecosystem, which consists of enterprises, universities and the government, and uses the differential game method to determine the optimal research and development efforts, the optimal benefits, the overall benefits and the core technology level of all parties involved in the higher education ecosystem under the three modes of the noncooperative research and development model, the cost-sharing mode and the collaborative research and development mode. The following conclusions are obtained through comparative analyses of the relevant equilibrium results under the three modes and numerical validation:

1. The cost-sharing mechanism can effectively improve the level of research and development efforts of enterprises and universities, the research and development benefits for all participants in the higher education ecosystem, and the level of benefits for the higher education ecosystem as a whole.
2. The degree of research and development effort of each of the higher education ecosystems under the collaborative research and development model is optimal for the overall benefit of the higher education ecosystem as well as the level of core technology, provided that the participation constraints of each of the higher education ecosystems are met at the same time;
3. Regardless of the R&D mode chosen by the participating subjects of the higher education ecosystem, the higher the R&D capability coefficient and the higher the R&D revenue coefficient are, the greater the R&D efforts of each participant and the greater the technological level of the higher education ecosystem. The revenue allocation coefficients of the enterprises and universities have a differentiated impact on the different participating subjects under different R&D models.

### Theoretical implications

1. Using differential game theory, a linkage model of antecedent conditions for technological innovation in higher education ecosystems based on a digital perspective is constructed, which expands the application context and research scope of higher education ecosystems and enriches the antecedent theoretical framework of BMI. Differential game theory has shown good insight and revelation in management. However, the literature on its application in the context of higher education ecosystems still needs to be reviewed. This study introduces differential game theory to the research field of technology R&D innovation in higher education ecosystems and constructs models of the noncooperative mode, cost-sharing mode, and collaborative mode, which can achieve organic integration and linkage analysis of different R&D modes of the participating subjects and respond to scholars’ call for the adoption of a new methodology to discuss the dynamic evolution process and paths of higher education ecosystems to promote the sustainable development of higher education ecosystems [30].
2. The necessary conditions for technological innovation in higher education ecosystems are explored using model-building and numerical simulation methods. This provides a new understanding of the complex causal relationship between higher education ecosystems and technological R&D innovation at a finer granularity. Based on the analysis of certain assumptions, this study comprehensively considered the equilibrium results of the participating subjects in the noncooperative R&D mode, cost-sharing mode, and collaborative mode, paying attention to how the assumed conditions acted on the innovation of the higher education ecosystem’s technology R&D mode through a complex causal mechanism and portrayed the technological innovation situation of the higher education ecosystem from a more holistic and systematic point of view. In addition, based on the influence of multiple variables on the subjects of higher education ecosystems and ecosystem benefits, this paper further identifies the differences in the strategic choices of different subjects in the evolutionary model of innovation equilibrium, which provides an important reference for ecosystem development, in response to scholars’ analysis of the influencing elements in the process of the dynamic evolution of higher education ecosystems [31].

### Management implications

1. The government should focus on the continuous construction of organizational resources and capabilities. According to the simulation results, the government’s capacity is always at a higher level in the R&D model, which promotes the technological innovation level of the higher education ecosystem. Therefore, the government should take into full consideration the differences in R&D capabilities and contributions between enterprises and universities; formulate scientific and practical cost-sharing guidelines; establish a multilevel cost-sharing mechanism for capital, personnel, and technology; and share R&D costs as much as possible within a reasonable range for enterprises and universities to motivate them to put in more R&D efforts. Therefore, enterprises with different resource endowments need to actively examine the external environment, identify R&D modes that match their resource endowments, and actively carry out technological innovation activities in the higher education ecosystem.
2. Universities should endeavor to increase their centrality in the higher education ecosystem and, simultaneously, choose to embed themselves in different relational networks, considering the specific context. On the one hand, high-level technological innovation networks play a universal role in promoting R&D model innovation. Therefore, universities should actively position themselves in the higher education ecosystem and endeavour to enhance communication with other participating subjects. On this basis, relying on their advantageous position in the higher education ecosystem, they can absorb and integrate the resources needed for R&D model innovation and then play the role of universities in promoting technological change, business innovation, and value creation.
3. The critical role of promoting the deep coupling and synergy of strategies, organizations, and resources in the higher education ecosystem and building a unified community of interest should likewise be noted. The government should play a leading role in actively opening up various innovation resources, such as design and R&D capabilities, instrumentation, test origins, etc., to enterprises and colleges and universities and strengthening support for their R&D and innovation. Colleges and universities should give full play to their advantages in basic research, cooperate with the government’s technological strategy, explore the underlying scientific principles purposely, and develop prototype systems with strong applicability and easy transformation. Enterprises should take the initiative in integrating into the government’s industrial chain, and conducting technological research and prototype R&D in response to enterprises should take the initiative to integrate into the government industrial chain and carry out technical research and prototype research and development in response to the government’s supporting technology needs. Specifically, they can combine the resource capacity and characteristics of the higher education ecosystem, choose the innovation and change paths that suit them, and propose novel and promising R&D model innovation programs at the right time.

## Research gaps and future prospects

To facilitate the solution, the model constructed in this paper contains only enterprises, universities and the government, and the relevant parameters have been simplified to some extent. Therefore, subsequent research can be improved in the following four aspects: (1) removing the research assumption that the parameters in the model are independent of time and considering the numerical solution of the model under dynamic parameter conditions; (2) considering the government’s influence mechanism on collaborative research and development in higher education ecosystems; (3) exploring the distribution of incremental benefits in higher education ecosystems under the collaborative cooperative research and development model; and (4) considering the interference of stochastic factors and establishing a stochastic differential game model to study collaborative research and development in higher education ecosystems.
